# Overcoming doxorubicin resistance in triple-negative breast cancer using the class I-targeting HDAC inhibitor bocodepsin/OKI-179 to promote apoptosis

**DOI:** 10.1186/s13058-024-01799-5

**Published:** 2024-03-01

**Authors:** Stephen G. Smoots, Anna R. Schreiber, Marilyn M. Jackson, Stacey M. Bagby, Adrian T A. Dominguez, Evan D. Dus, Cameron A. Binns, Morgan MacBeth, Phaedra A. Whitty, Jennifer R. Diamond, Todd M. Pitts

**Affiliations:** Department of Medicine, Division of Medical Oncology, University of Colorado Anschutz Medical Campus, 12801 East 17th Avenue MS8117, Aurora, CO 80045 USA

**Keywords:** Triple-negative breast cancer, Doxorubicin, Histone deacetylase inhibitor

## Abstract

**Background:**

Triple-negative breast cancer (TNBC) is an aggressive breast cancer subtype with a poor prognosis. Doxorubicin is part of standard curative therapy for TNBC, but chemotherapy resistance remains an important clinical challenge. Bocodepsin (OKI-179) is a small molecule class I histone deacetylase (HDAC) inhibitor that promotes apoptosis in TNBC preclinical models. The purpose of this study was to investigate the combination of bocodepsin and doxorubicin in preclinical TNBC models and evaluate the impact on terminal cell fate, including apoptosis and senescence.

**Methods:**

TNBC cell lines were treated with doxorubicin and CellTiter-Glo was used to assess proliferation and determine doxorubicin sensitivity. Select cell lines were treated with OKI-005 (in vitro version of bocodepsin) and doxorubicin and assessed for proliferation, apoptosis as measured by Annexin V/PI, and cell cycle by flow cytometry. Immunoblotting was used to assess changes in mediators of apoptosis, cell cycle arrest, and senescence. Senescence was measured by the senescence-associated β-galactosidase assay. An MDA-MB-231 xenograft in vivo model was treated with bocodepsin, doxorubicin, or the combination and assessed for inhibition of tumor growth. shRNA knockdown of p53 was performed in the CAL-51 cell line and proliferation, apoptosis and senescence were assessed in response to combination treatment.

**Results:**

OKI-005 and doxorubicin resulted in synergistic antiproliferative activity in TNBC cells lines regardless of p53 mutation status. The combination led to increased apoptosis and decreased senescence. In vivo, the combination resulted in increased tumor growth inhibition compared to either single agent. shRNA knock-down of p53 led to increased doxorubicin-induced senescence that was decreased with the addition of OKI-005 in vitro.

**Conclusion:**

The addition of bocodepsin to doxorubicin resulted in synergistic antiproliferative activity in vitro, improved tumor growth inhibition in vivo, and promotion of apoptosis which makes this a promising combination to overcome doxorubicin resistance in TNBC. Bocodepsin is currently in clinical development and has a favorable toxicity profile compared to other HDAC inhibitors supporting the feasibility of evaluating this combination in patients with TNBC.

**Supplementary Information:**

The online version contains supplementary material available at 10.1186/s13058-024-01799-5.

## Introduction

Triple-negative breast cancer (TNBC) is an aggressive subtype of breast cancer that is characterized by a lack of estrogen receptor (ER), progesterone receptor (PR), and human epidermal growth factor receptor (HER2) amplification [1]. TNBC accounts for 15–20% of all breast cancer cases and is associated with a more aggressive clinical course [2]. Patients with TNBC have a greater risk of early recurrence, distant metastasis, and overall shorter survival compared to patients with other breast cancer subtypes [2]. While progress has been made recently with the development of effective targeted therapies for a subset of patients with TNBC, chemotherapy remains a mainstay of treatment. Anthracycline-based chemotherapy regimens continue to be standard of care for most patients receiving neoadjuvant chemotherapy for TNBC in the curative setting [3]. For patients who do not experience a pathologic complete response following neoadjuvant therapy, the risk of metastatic recurrence remains high [4]. There remains a clinical need to understand resistance mechanisms to chemotherapeutics, including doxorubicin, in TNBC and to develop novel combinations to overcome this resistance and increase clinical benefit.

Doxorubicin (DOX) is a topoisomerase II inhibitor that interferes with DNA repair via DNA intercalation, halting DNA replication and inducing cell apoptosis, autophagy, or senescence [5, 6]. Treatment with doxorubicin, like many other cancer therapies, can lead to therapy-induced senescence (TIS) in a subset of cancer cells [7–9]. Senescence is a cellular phenomenon where cells irreversibly enter an arrested state, losing their ability to respond to growth stimuli while remaining metabolically active [10]. While some studies indicate that permanent cessation of cell division through induction of senescence can have anti-cancer activity, senescent cells secrete pro-tumorigenic growth factors as part of the senescence-associated secretory phenotype (SASP) which can alter the tumor landscape and promote tumor progression [8–13]. We have previously demonstrated an association between therapy-induced senescence and resistance to Aurora kinase inhibitors in preclinical models of TNBC [14]. This observation supports the hypothesis that the promotion of apoptosis may result in increased efficacy of anti-cancer therapies in TNBC.

*TP53* is the most commonly mutated gene in TNBC with an occurrence of approximately 85% [15]. p53 is responsible for the activation of several essential genes involved in DNA repair, cell cycle arrest, senescence, and apoptosis in response to DNA damage. Mutations in p53 are heterogeneous, but the majority are missense mutations that occur in the DNA binding domain spanning 190 codons [16]. Within the 190 codon region, these mutations occur more frequently at 8 specific codons termed the “hot spot” (R175, G245, R248, R249, R273, R282, V157F, Y220C) [16]. Certain p53 mutations can limit the cancer cell’s ability to induce apoptosis under DNA damage, allowing for continued proliferation or senescence. Mutations in the p53 gene in breast cancer is associated with differential responses to doxorubicin [17]. Mutations affecting the L2 or L3 DNA binding domains of the p53 gene, that render the TP53 protein transcriptionally inactive, or cause loss-of-function are implicated in resistance to doxorubicin in TNBC. However, other mutations in the p53 gene in TNBC may result in gain-of-function [18]. These mutations can lead to the promotion of anti-apoptotic pathways and impair autophagy. Given the role of doxorubicin-induced senescence in drug resistance, efforts to shift terminal cell fate from senescence to apoptosis may improve the efficacy of doxorubicin.

Global dysregulation of histone acetylation has been implicated in TNBC tumorigenesis and progression [19]. Histone deacetylases (HDACs) enzymatically remove acetyl groups from lysine residues on amino-terminal histone tails, condensing chromatin and silencing gene expression [20]. HDAC inhibitors can target these epigenetic modifications to permit histone acetylation, promoting uncoiled DNA and increased gene transcription [21]. Notably, histone deacetylase inhibitors effectively promote apoptosis in some preclinical models by modulating the expression of genes integral to both the extrinsic and intrinsic apoptotic pathways [22].

Bocodepsin (OKI-179) is a novel, orally bioavailable class 1-targeting HDAC inhibitor with promising anti-cancer activity in preclinical models of TNBC [21]. Both bocodepsin and OKI-005 are prodrugs that rapidly metabolize into the active metabolite OKI-006 [21]. Bocodepsin was further optimized for in vivo work as orally bioavailable. Recent preclinical studies demonstrated that bocodepsin could overcome PD1 blockade in resistant B-cell lymphomas [23]. Here we report the combination of doxorubicin and bocodepsin may be an effective therapeutic strategy for overcoming DOX resistance in TNBC. This combination resulted in decreased proliferation, increased apoptosis, and decreased senescence in TNBC cell lines, regardless of p53 status. The combination further demonstrated anti-proliferative and anti-cancer effects in mouse models of TNBC.

## Methods

### Cell lines and reagents

Human TNBC cell lines MDA-MB-436, MDA-MB-231, MDA-MB-157, HCC1395, HCC1937, HCC38, HCC1187, HCC1143, HCC1395, Hs 578T, and BT549 were obtained from American Type Culture Collection (ATCC). CAL-85–1, CAL-51, CAL-148, CAL-120, and HDQ-P1 were obtained from the Deutsche Sammlung von Mikroorganismen und Zellkulturen GmbH (DSMZ). BT20, HCC70, and MDA-MB-468 were obtained from the University of Colorado Cancer Center Cell Technologies Core, (UCCC; Aurora, Colorado, USA). shRNA cell lines CAL-51 SCR and CAL-51 P53-10 were created as previously described [14]. All cell lines were cultured in Corning Dulbecco’s Modified Eagle Media (DMEM), supplemented with 10% FBS (Atlas Biologicals), 1% Normocin (InvivoGen), 1% penicillin/streptomycin (Gibco) and 1% minimum essential media nonessential amino acids (Corning). Cell lines were continuously maintained in a 37° incubator with 5% CO_2_ and tested for mycoplasma regularly. For in vitro studies, OKI-005 was obtained from OnKure, Inc. (Boulder, CO, USA) and dissolved in DMSO to a final working concentration of 10 mM and doxorubicin was purchased from Selleck Chemicals (Houston, TX, USA) and was dissolved in DMSO and stored at a 10mM stock. For in vivo studies, bocodepsin was obtained from OnKure Inc. and dissolved in a 5:1 solution of citric acid (0.1 M) and sodium citrate (0.1 M) and sonicated at room temperature for one hour. OKI-005 is the cell active predecessor of OKI-179 and due to drug properties, it is preferred for use in vitro. OKI-179 is the optimized prodrug for in vivo and human use. The development of OKI-179 and OKI-005 as prodrugs of the active metabolite OKI-006 is further described in Diamond et al., Preclinical development of the class I selective HDAC inhibitor OKI-179, Molecular Cancer Therapeutics 2022 [21]. Doxorubicin was purchased from the University of Colorado Research Pharmacy and diluted 1:5.8 in sterile saline (Aurora, CO).

### Viability and proliferation assays

Briefly, cells were seeded at 8,000 cells per well in a white-walled 96-well plate prior to exposure to doxorubicin (0.0625 µM, 0.125 µM, 0.25 µM, 0.5 µM. 1 µM, 2 µM), OKI-005 (0.1 µM, 0.2 µM, 0.4 µM) or the combination. Plates were read on the BioTek Synergy H1 plate reader following 72 h of drug treatment and replicated three times. The CellTiter-Glo Luminescent Cell Viability Assay (Promega, Madison, WI) was used to quantify cell viability using the manufacturer’s instructions. Synergy scores using the average of three replicates were calculated using Synergy Finder^+^ [24]. To further verify anti-proliferative effects, cells were seeded at 4,000 cells per well in a 96-well plate. After 24 h of cell adhesion, 0.5 µM doxorubicin, 0.1 µM OKI-005, or combination were added to the cells and proliferation for 72 h was measured using the Agilent BioTek Biospa live cell analysis system. Images were taken every 4 h, and live cells were calculated using Agilent BioTek’s high contrast cell counting. Three independent replicates were performed.

### Annexin-V-FITC/PI and Annexin-V-APC/PI

Cells were seeded in 6-well plates and allowed to adhere for 24 h. Cells were then dosed with doxorubicin (0.5 µM), OKI-005 (0.2 µM), or the combination for 24 and 48 h. Cells were then trypsinized, neutralized with media, and washed with PBS. Cells were transferred to flow cytometry tubes and resuspended with Annexin-V-FITC and PI (Cell Signaling Technology, Danvers, MA, #6592). Due to the GFP-tagged constructs in CAL-51 SCR and CAL-51 p53-10 cell lines, Annexin-V-APC and PI were used (Biolegend, San Diego, CA, #640,919). Samples were run and analyzed by the UCCC Flow Cytometry Shared Resource.

### Cell cycle analysis

Cells were seeded at 150,000 cells per well in a 6-well plate and allowed to adhere for 24 h. Doxorubicin (0.5 µM), OKI-005 (0.2 µM), or the combination was added for 24 h. Cells were then trypsinized and washed with PBS prior to 70% methanol fixation on ice. Fixed cells were left on ice for at least 15 min before 50 µg/ml RNase (Thermo Scientific, Waltham, MA) and 1 mg/ml PI (Invitrogen, Waltham, MA) were added. After 30 min at room temperature, cells were run on the BioRad ZE5 cytometer and analyzed via Modfits.

### Immunoblotting

Cells were seeded in 100 mm dishes and allowed to adhere for 24 h at 37°. Cells were then exposed to 0.5 µM doxorubicin, 0.2 µM OKI-005, or the combination. Cells were lysed in Pierce RIPA Buffer with 1X Halt Protease and Phosphatase Inhibitor Cocktail then sonicated. After sonication, total protein was measured using the Pierce BCA Protein Assay Kit. 15–40 µg of total protein was loaded on an Invitrogen 4–12% Bis-Tris Midi 1.0 mm gradient gel, electrophoresed, and then transferred to a nitrocellulose membrane using an Invitrogen Power Blotter Station. Membranes were blocked in casein blocking buffer for 1 h and incubated overnight at 4° with the following primary antibodies from Cell Signaling Technology: p-RAD50 (#14,223), p-histone H2A.X (#9718), cleaved caspase-3 (#9661), p53 (#48,818), p21 (#2946), p16 (#80,772), BCL-XL (#2764), acetyl-histone H3 (#9649), BAX (#89,477), cyclin B1 (#4135), and actin (#58,169, #4970). The p53 antibody selected (DO-7) binds to the N-terminus and can recognize both p53 wild-type and certain p53 mutants. Following primary antibody incubation, membranes were washed thrice in TBS-Tween (0.1%) for five minutes before an hour incubation at room temperature with the appropriate secondary anti-rabbit IgG (H + L) (DyLight 680 Conjugate, #5470) and/or anti-rabbit IgG (H + L) (DyLight 800 4X PEG Conjugate, #5151) at a 1:10,000 dilution (Cell Signaling Technology, Danvers, MA). After 3 additional washes, blots were imaged using the Odyssey Infrared Imaging System (LI-COR Biosciences, Lincoln, NE). Three independent replicates were performed to confirm the findings.

### Senescence associated β-galactosidase

For in vitro, cells were seeded in 6-well plates and allowed to adhere for 24 h at 37° prior to treatment with doxorubicin (0.1 µM, 0.5 µM) and OKI-005 (0.2 µM) for 6 days. Cells treated with a single drug were plated at 100,000 cells per well while cells dosed with both drugs were plated at 200,000 cells per well to account for drug effects. Untreated cells were plated at 25,000 cells per well to reduce overgrowth. Following drug treatment, cells were fixed and stained using the senescence β-galactosidase staining kit from Cell Signaling Technologies (#9860). Prior to imaging, cells were incubated for 10 min with 300 nM DAPI (Thermo Scientific, Waltham, MA). For in vivo, tissue was collected from all sample groups on day 36 and frozen in OCT. OCT samples were cut on a cryostat at 5 μm and fixed and stained with the previous kit described. Samples were then counterstained with eosin for 2 min, rinsed with water, and mounted with Permount (Thermo Scientific, Waltham, MA). An Olympus iX83 microscope was utilized to obtain representative images at 20X magnification. β-galactosidase expression was quantified by measuring β-galactosidase pixel density with ImageJ.

### Tumor xenograft model

All murine studies were performed following the approval of University of Colorado Anschutz Medical Campus Institutional Animal Care and Use Committee. Female Athymic Nude Mice (Hsd: Athymic Nude-Foxn1nu) aged 12–13 weeks were purchased from Envigo (Indianapolis, IN). MDA-MB-231 cells were collected during the logarithmic growth phase and injected subcutaneously at 5 × 10^6^ cells on each flank under isoflurane anesthesia. The injection was 100ul per flank, consisting of a 1:1 ratio of DMEM media and cultrex (Cultrex PathClear BME, Type 3 from Bio-Techne). Treatment with either vehicle, 1.5 mg/kg doxorubicin IP QW, 80 mg/kg bocodepsin PO QD, or the combination was started when tumors reached an approximate average volume of 100mm^3^. Mice were monitored daily for toxicity, and weight and tumor volume were collected twice per week using a digital scale, calipers, and Studylog version 3.1.399.23 (San Francisco, CA). The following equation was used to calculate tumor volume: volume = (length x width^2^) x 0.52. Formalin-fixed paraffin-embedded tumor was collected at the end of study and stained for H&E, Ki67, and cleaved-caspase 3 by the UCCC Pathology Shared Resource.

### Statistical analysis

Graph Pad Prism 10 was used for all statistical analysis. Specific tests applied are noted in figure legends. Specific growth rate was calculated as previously described [25]. *P* values ≤ 0.05 were considered statistically significant.

## Results

### Doxorubicin has enhanced antiproliferative activity in combination with OKI-005

**Fig. 1:**
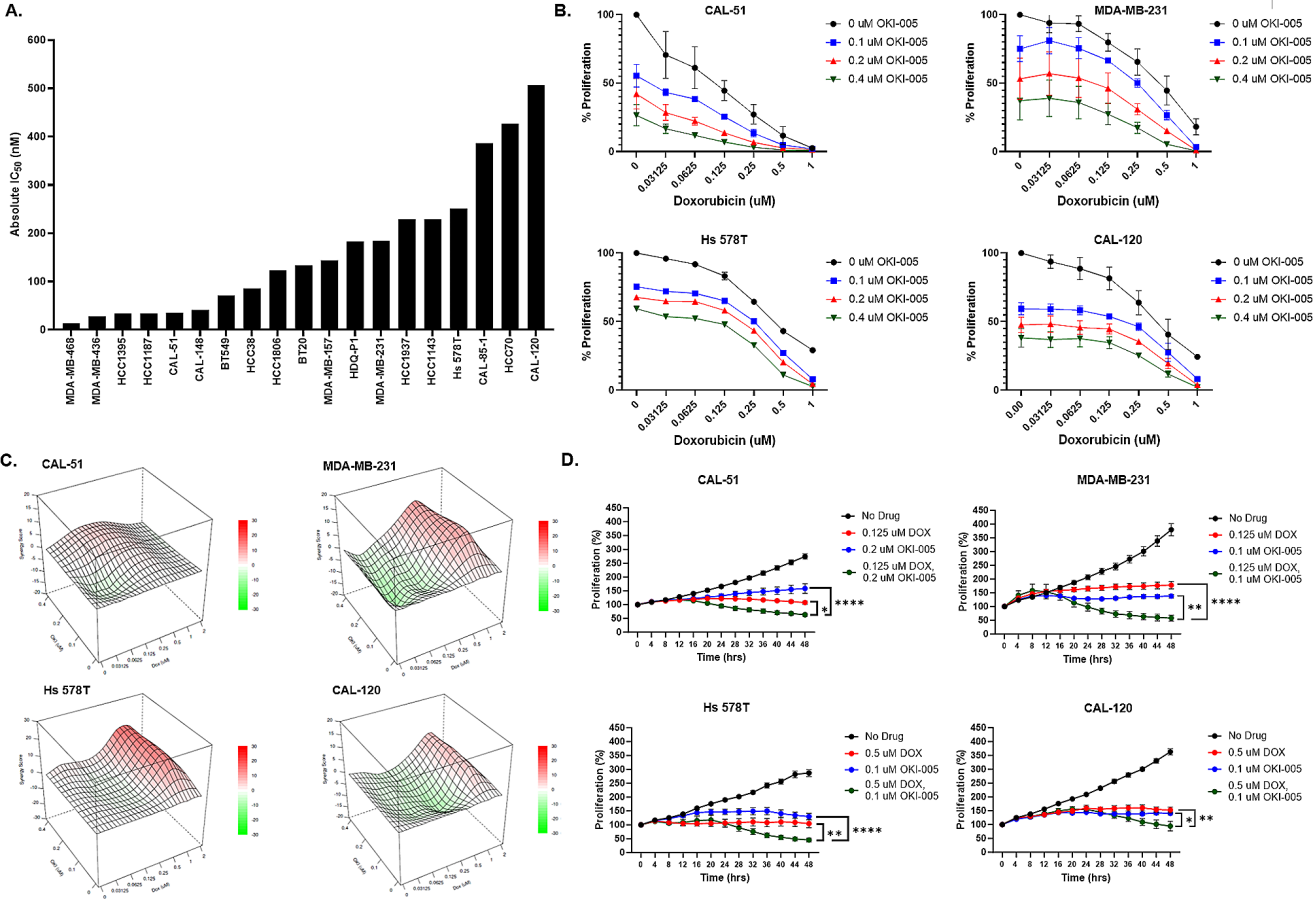
**(A)** Sensitivity of p53 mutated or wild-type TNBC cell lines to doxorubicin. IC_50_ values were calculated by CellTiter-Glo following 72 h doxorubicin exposure. **(B)** Effects of DOX alone or in combination with OKI-005 for CAL-51 (wild-type p53), MDA-MB-231 (mutant p53), Hs 578T (mutant p53), and CAL-120 (mutant p53) as measured by CellTiter-Glo after 72 h drug exposure. **(C)** Synergistic effects of DOX and OKI-005. Bliss scores were calculated from the data in Fig. 1B using Synergy Finder^+^. **(D)** Anti-proliferative effects of DOX in combination with OKI-005 measured by live cell imaging for 48 h. Statistical analysis comparing single agent to combination was determined by ordinary one-way ANOVA with Tukey correction (* = *p* < 0.05)

We first screened multiple p53 mutated and p53 wild-type (CAL-51) cell lines to determine their sensitivity/resistance to DOX. IC_50_ values were calculated using CellTiter-Glo after 72 h doxorubicin exposure, where the IC_50_ values ranged from 13 to 507 nM (Fig. 1A, Supplemental 1A). The 4 cell lines CAL-51 (35 nM), MDA-MB-231 (184 nM), Hs 578T (251 nM), and CAL-120 (507 nM) were selected for further examination in combination with OKI-005. CAL-51 was selected because it is more responsive to DOX and p53 wild-type, while MDA-MD-231 and Hs 578T are moderately responsive to DOX. CAL-120 was selected as the least responsive to DOX. To characterize the combination effect of DOX and OKI-005, CellTiter-Glo was performed to determine cell viability after 72 h (Fig. 1B). CellTiter-Glo quantifies ATP present in cultured cells and synergistic responses can be calculated using BLISS. We observed synergistic combination effects at various drug combinations in all four of the cell lines that had a bliss value range between 10 and 22 (Fig. 1C). To further examine the anti-proliferative effects, we used live cell imaging for 48 h with Agilent BioTek’s high contrast cell counting to further verify the anti-proliferative effects on the cell lines. We observed significance from both the single agents DOX and OKI-005 to the combination (Fig. 1D).

### Doxorubicin in combination with OKI-005 generates increased apoptosis with altered cell cycle arrest

**Fig. 2:**
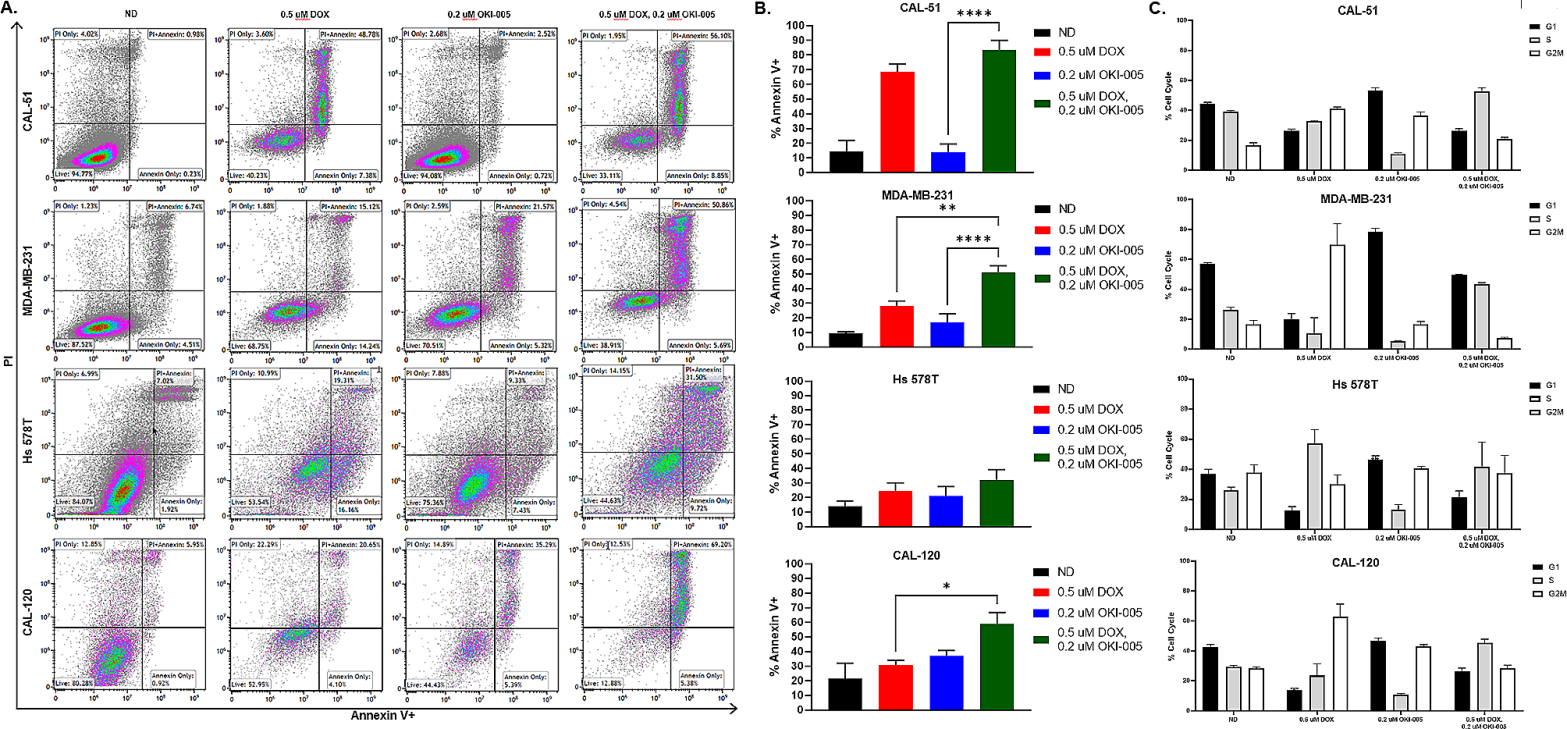
**(A)** Pro-apoptotic effects of DOX in combination with OKI-005. Representative plots of Annexin V/ PI in the cell lines CAL-51 (wild-type p53), MDA-MB-231 (mutant p53), Hs 578T (mutant p53), and CAL-120 (mutant p53) cell lines after 48 h of treatment with DOX (0.5 µM), OKI-005 (0.2 µM), or combination as measured by flow cytometry. **(B)** Statistical analysis of apoptosis via total Annexin V. Ordinary one-way ANOVA with Tukey correction comparing single agent to combination (* = *p* < 0.05, ** = *P* < 0.01; *** = *P* < 0.001). A trend (*p* = 0.058) for CAL-120 OKI-005 compared with the combination was observed. **(C)** Cell cycle analysis of DOX and OKI-005 for 24 h measured by flow cytometry via PI

Based on the cell viability results in Fig. 1, we looked at the induction of apoptosis with DOX and OKI-005 using the Annexin-V/PI assay. Exposure of cell lines to the combination for 48 h resulted in greater apoptosis as measured by Annexin-V when compared to single agent DOX or OKI-005 (Fig. 2A, B). In the CAL-51 cell line, DOX significantly enhanced the apoptotic effects of OKI-005 in combination. MDA-MB-231 displayed a significant increase for both single agents, DOX and OKI-005, as compared to the combination. We observed no significant increase in apoptosis for the cell line Hs 578T at 48 h but did see significance between DOX and the combination for CAL-120. For the DOX-sensitive cell line CAL-51, the combination significantly increased apoptosis compared to both single agents after 24 h of drug exposure (Supplemental 1B). To determine if the apoptotic effects seen in the combination were caused by altered cell cycle dynamics, flow cytometry was performed after 24 h of drug treatment. Notably, 0.5 µM DOX generated a G2M arrest while 0.2 µM OKI-005 caused an G1 phase arrest in all cell lines. The combination of DOX and OKI-005 resulted in an S phase arrest.

### Induction of apoptosis is mediated by the intrinsic pathway via BCL-2 family members

**Fig. 3:**
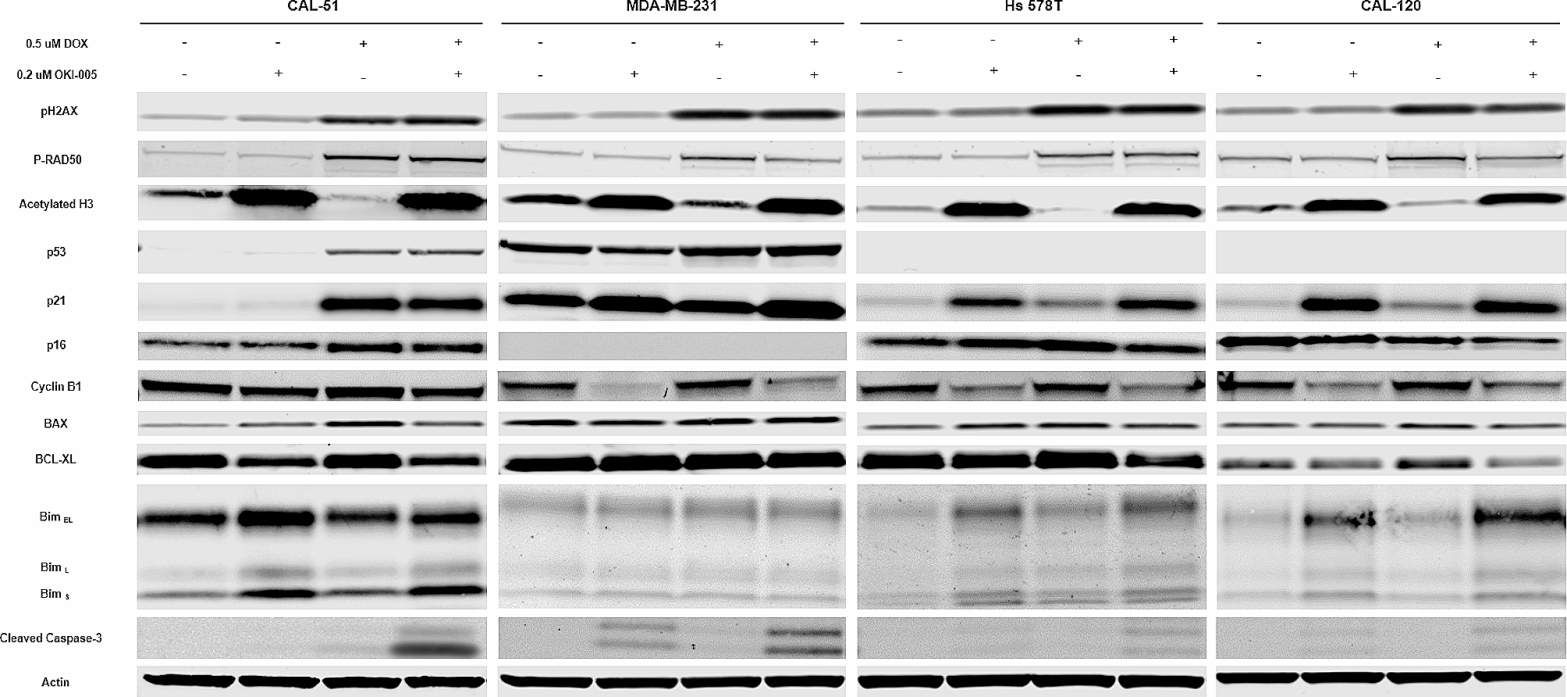
Effects of DOX and OKI-005 on downstream effectors of DNA damage, apoptosis, and cell cycle. Cells were treated for 24 h with DOX (0.5 µM), OKI-005 (0.2 µM), or combination

To further investigate mediators of apoptosis, cell cycle checkpoints, and senescence, immunoblots were performed 24 h post drug exposure (Fig. 3). An increase in the DNA damage markers P-H2AX and P-RAD50 was observed following DOX treatment, which was maintained in combination. OKI-005 caused a marked increase in acetylated H3 as observed previously [21]. p53 increased in CAL-51 and MDA-MB-231 from DOX, but no expression was observed for Hs 578T and CAL-120. An increase in the expression of p21 was observed in MDA-MB-231, Hs 578T, and CAL-120 for all drug treatment groups, with the greatest increase in OKI-005 and combination. OKI-005 decreased expression of cyclin B1 in all cell lines which was further maintained in the combination. The senescent marker p16 was slightly increased from DOX in CAL-51 and Hs 578T. BCL-XL, an anti-apoptotic protein, was decreased in combination while the pro-apoptotic protein BIM was increased for CAL-51, Hs 578T, and CAL-120. The pro-apoptotic effects appear to be mediated via caspase-3, as we observed elevated cleaved caspase-3 in combination for all cell lines (Supplemental 1 C).

### Doxorubicin-induced senescence is mitigated by OKI-005

**Fig. 4:**
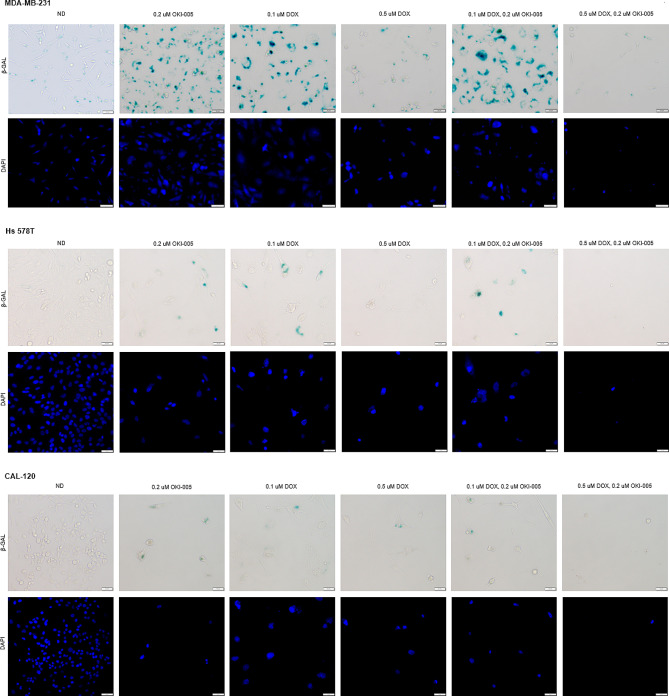
Senescence-associated β-galactosidase staining of MDA-MB-231, Hs 578T, and CAL-120 after 6 days of drug treatment. DAPI staining portrays the nucleus. Representative images were taken at x20 magnification. Scale 50 μm

Therapy-induced senescence (TIS) has previously been hypothesized as a mechanism of DOX-induced resistance. To determine if the combination of DOX and OKI-005 reduced senescence, we performed the senescence-associated β-galactosidase (SA-β-gal) assay after 6 days of drug exposure (Fig. 4). We observed the largest increase of senescence (SA-β-gal positive) in MDA-MB-231, Hs 578T, and CAL-120, at 0.1 µM DOX. Other senescence features such as enlarged cell morphology and nuclei coincided with the positive SA-β-gal we observed. At 0.5 µM DOX, senescence was seen in MDA-MB-231 and CAL-120, but not Hs 578T. OKI-005 alone also generated senescent cells. Furthermore, the combination of OKI-005 with 0.5 µM DOX decreased senescence, but not at 0.1 µM DOX for all cell lines.

### Reduced senescence in vivo corresponds with favorable inhibition of tumor growth

**Fig. 5:**
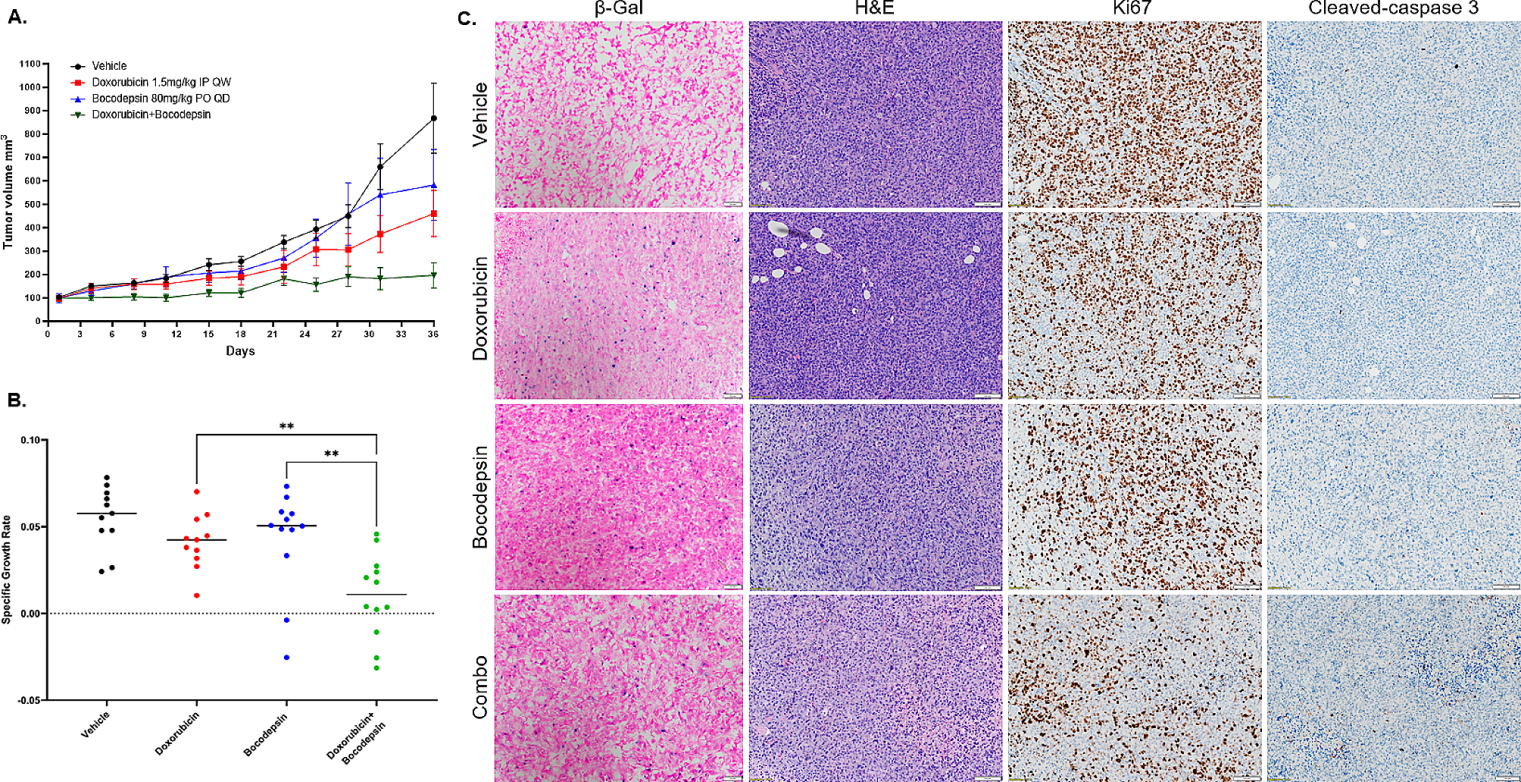
**(A)** MDA-MB-231 xenograft model athymic nude mice treated with vehicle, doxorubicin, bocodepsin, or combination for 36 days. **(B)** Specific growth rates at day 36 depicting overall tumor growth inhibition (** = *P* < 0.01). **(C)** Senescence-associated β-galactosidase staining of frozen tissue collected at day 36. Scale 50 μm. H&E, Ki67, and cleaved-caspase 3 staining of formalin-fixed tissue collected at day 36. Representative images were taken at x20 magnification. Scale 100 μm

To confirm that the antiproliferative activity observed in vitro can be clinically relevant, we selected the cell line MDA-MB-231 for a xenograft model based on its resistance to doxorubicin and consistent growth patterns in vivo. The combination treatment resulted in relatively minimum tumor growth as compared to either doxorubicin or bocodepsin (Fig. 5A). Specific growth rates showed the combination significantly decreased from both doxorubicin (*p* = 0.0016) and bocodepsin (*p* = 0.0068) after 36 days (Fig. 5B). Senescence-associated β-galactosidase staining further revealed that doxorubicin and bocodepsin both increase senescence in vivo, whereas the drug combination limited senescence emergence (Fig. 5C, Supplemental 1). Decreased Ki67 and increased cleaved-caspase 3 further correlated with decreased senescence formation (Fig. 5C). While we did observe some drug toxicity based on body weight, body condition, and general health after 36 days of treatment, the combination was overall well tolerated (Supplemental 2 A).

### Doxorubicin in combination with OKI-005 retains effectiveness with loss of wild-type p53

**Fig. 6:**
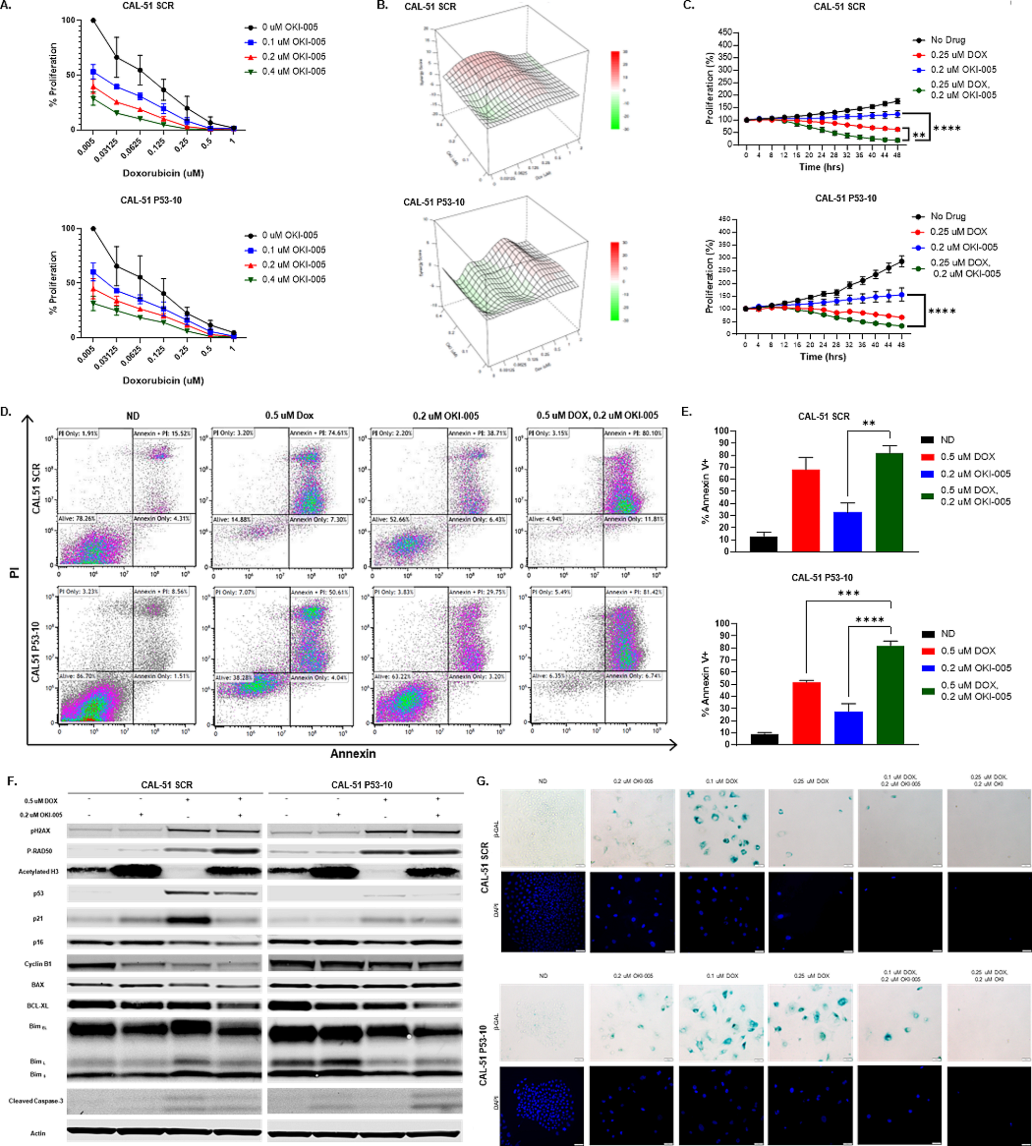
**(A)** Effects of p53 knockdown on DOX and OKI-005 sensitivity measured by CellTiter-Glo after 72 h drug exposure. **(B)** Bliss scores calculated from the data in Fig. 6A using Synergy Finder^+^. **(C)** Anti-proliferative effects of DOX (0.25 µM), OKI-005 (0.2 µM) or combination measured by live cell imaging for 48 h. **(D)** Pro-apoptotic effects measured by Annexin V/ PI after 48 h of DOX (0.5 µM), OKI-005 (0.2 µM), or combination. **(E)** Statistical analysis of apoptosis via total Annexin V. Ordinary one-way ANOVA with Tukey correction comparing single agent to combination (** = *P* < 0.01; *** = *P* < 0.001; **** = *P* < 0.001). **(F)** Effects of DOX and OKI-005 on downstream effectors of DNA damage, apoptosis, and cell cycle. Cells were treated for 24 h with DOX (0.5 µM), OKI-005 (0.2 µM), or combination. **(G)** Senescence-associated β-galactosidase staining of MDA-MB-231, Hs 578T, and CAL-120 after 6 days of drug treatment. DAPI staining portrays the nucleus. Representative images were taken at x20 magnification. Scale 50 μm

We developed a p53 knockdown (KD) cell line (CAL-51 P53-10) and a control (CAL-51 SCR) using shRNA to observe the direct effects of p53 on DOX resistance. rtPCR verified the KD efficiency of p53 (Supplemental 2B). CellTiter-Glo after 72 h of drug treatment demonstrated no significant difference with loss of WT p53 (Fig. 6A). However, CAL-51 P53-10 was more synergistic in the drug combination at a higher DOX concentration (0.5 µM) than CAL-51 SCR. Proliferation as measured by live cell imaging demonstrated a significant difference between DOX and the combination for CAL-51 SCR, but not CAL-51 P53-10 (Fig. 6C**).** We also observed an increase in proliferation without drug treatment when p53 was knocked down. Apoptosis measured by annexin V after 48 h of drug treatment demonstrated a significant increase between OKI-005 and the combination for both CAL-51 SCR and CAL-51 P53-10. We further show knockdown of p53 decreases the apoptotic response generated from doxorubicin (Fig. 6D, E). After 24 h of drug treatment an increase in apoptosis was observed in the combination for both CAL-51 SCR and CAL-51 P53-10 (Supplemental 2 C). Knockdown of p53 showed an increase in p16 for the combination treatment when compared to CAL-51 SCR. BIM increased in CAL-51 SCR with DOX and decreased in CAL-51 P53-10 (Fig. 6F). Lastly, we looked at p53’s role in senescence as a possible mediator of DOX resistance. β-Gal staining after 6 days of drug treatment showed senescence in both CAL-51 SCR and CAL-51 P53-10 at low dose DOX (0.1 µM). However, at a higher dose of DOX (0.25 µM), there was noticeably more senescence in CAL-51 P53-10 than in CAL-51 SCR. The addition of OKI-005 to DOX decreased senescent cells regardless of p53 status (Fig. 6G, Supplemental 2D).

## Discussion

Doxorubicin resistance remains a major clinical challenge for TNBC patients [2]. Of particular interest, TP53 is mutated in nearly 80% of all TNBC as compared to 30% of all other breast cancer subtypes [15]. Since doxorubicin functions as a topoisomerase II inhibitor via DNA intercalation, mutated p53 is heavily speculated to contribute to doxorubicin-induced resistance [5, 17]. p53, a transcription factor triggered by DNA damage, is involved in the activation of multiple essential genes including DNA repair, cell cycle arrest, apoptosis, and senescence. Previous studies for HER2 + breast cancer have shown mutated p53 correlates with a decrease in the overall survival rate of metastatic breast cancer patients [26]. While neoadjuvant chemotherapy regimens including doxorubicin remain a mainstay treatment option for TNBC patients in the clinic, not all patients receive a pathological complete response. The purpose of this study was to evaluate the anti-cancer activity from the combination of doxorubicin and OKI-005/bocodepsin in TNBC. We confirmed doxorubicin treatment leads to senescence in resistant TNBC cells following treatment. We also confirmed the addition of OKI-005 to higher doses of doxorubicin leads to increased cell death, promotion of apoptosis, and decreased senescence. These findings were consistent in p53 wild-type and p53 mutated cell lines, as well as a xenograft mouse model.

Our lab previously showed Bocodepsin, a novel class I HDACi, to have pro-apoptotic and anti-proliferative effects for TNBC in vivo [21]. We demonstrate here that doxorubicin combined with bocodepsin can be a potential clinical treatment option for TNBC patients. Initial screening of the combination demonstrated synergistic effects in both p53 wild-type and p53 mutant TNBC cell lines, with evidence of decreased proliferation and increased apoptosis. We demonstrate the pro-apoptotic activity caused by the combination is mediated by BCL-2 family members. Previous studies have shown elevated levels of the BCL-XL coding gene, BCL2L1, correlates with a poor survival outcome for TNBC patients [27]. Doxorubicin increased the anti-apoptotic protein BCL-XL in the CAL-51, Hs 578T, and CAL-120 cell lines, while OKI-005 had antagonist effects for BCL-XL. The combination resulted in decreased BCL-XL, demonstrating the addition of OKI-005 can overcome potential doxorubicin-induced resistance. Another BCL-2 family member BIM was increased by OKI-005, which has previously been reported by other HDAC inhibitors [28]. Ultimately, the decrease of anti-apoptotic proteins and increase in pro-apoptotic proteins caused by the combination resulted in increased cleaved caspase-3. Interestingly, the combination generated no change in BCL-XL and BIM for MDA-MB-23, but still resulted in significant apoptosis as seen by an increase in annexin V and cleaved-caspase 3. This may be due to elevated mutant p53 losing its ability to activate BIM directly or indirectly [29–31]. We further showed the combination could reduce BCL-XL with loss of wild-type p53, demonstrating the pro-apoptotic abilities may be p53-independent. However, the loss of p53 limited BIM induction for the combination, possibly activating apoptosis through a different pathway.

Overexpression of cyclin B1 can initiate uncontrolled cell growth and has been linked to aggressive tumor behavior with increased lymphovascular invasion in breast cancer [32, 33]. Knockdown of cyclin B1 sensitizes chemotherapies, leading to decreased cell proliferation and increased apoptosis [34]. Our data is consistent with other studies reporting doxorubicin increases or retains cyclin B1 [35, 36]. We demonstrate OKI-005 decreases cyclin B1 expression, which is retained in combination with doxorubicin. Decreased cyclin B1 was retained in combination with both wild-type p53 and mutant p53. However, the knockdown of p53 mitigated the effects of OKI-005 on cyclin B1, signaling that p53 may be required to repress cyclin B1. While we further observed doxorubicin to cause a G2M arrest and OKI-005 a G1 arrest, we demonstrate here the combination arrests the cells in S phase. While the mechanism is still unclear for the S phase arrest, the combination ultimately inhibits proliferation, with reduced senescent cell formation and increased apoptosis.

Cellular senescence is an irreversible growth arrest caused by different types of cellular stress [10, 37]. The role of senescence as a terminal cell fate following cancer therapy has evolved over the last decade. While in some situations, senescence may be a favorable cell fate (CDK4/6i) we have shown in multiple preclinical models that senescent cells are present at times of resistance [14]. Senescence caused by extensive doxorubicin-induced DNA damage has been hypothesized as a potential resistant mechanism in TNBC. Current literature remains contradictory regarding senescence exploiting cancer survival and progression, but evidence shows shifting terminal cell fate from senescence to the desired apoptotic response is beneficial [7–12]. p53 is a well-known mediator of senescence, but some literature has demonstrated p53 can also suppress senescence [37–39]. We illustrate the latter where loss of wild-type p53 via shRNA increased senescence upon doxorubicin treatment, which correlated with reduced apoptosis. We also show OKI-005 induced senescence as a single therapeutic agent, similar to other HDAC inhibitors [40]. We demonstrate a higher doxorubicin concentration is required with OKI-005 to reduce senescent cell formation, which is most likely due to adequate buildup of apoptotic-inducing DNA damage. We also confirmed doxorubicin greatly increased senescence in vivo, reinforcing senescence as a possible mechanism of doxorubicin resistance. Indeed, Bocodepsin diminished doxorubicin-induced senescence in vivo, but we did not observe the complete loss of senescence from the combination as we did in vitro.

While we provide promising preclinical data here for the combination of bocodepsin with doxorubicin, bocodepsin is also currently being investigated in combination with binimetinib in an ongoing phase 1b/2 clinical trial. This trial is based on a body of literature supporting the role of HDAC3 inhibition in potentiating the activity of MEK inhibitors in RAS pathway-altered tumors [41, 42]. In patients with advanced NRAS-mutated melanoma previously treated with immune checkpoint inhibitors, the preliminary overall response rate was 38% with bocodepsin and binimetinib compared historically to 16% for binimetinib alone in the NEMO trial [43, 44]. Other ongoing investigations of bocodepsin/OKI-005 are focused on combination strategies with immune checkpoint inhibitors. In B-cell lymphoma, bocodepsin in combination with anti-PD1 overcame PD1-blockade resistance by increasing tumor immunogenicity [23]. Similar results were observed in a murine colorectal tumor model, where an enhanced response was observed when treatment of bocedepsin was stopped prior to anti-PD1 treatment [45]. While the preliminary data for bocodepsin is promising, further investigation of doxorubicin in combination with bocodepsin for TNBC is warranted based off our preclinical results.

## Conclusion

In conclusion, we report the combination of doxorubicin with bocodepsin may be a novel therapeutic option for TNBC. Since p53 is mutated in a majority of all TNBC patients (85%), drug combinations as seen here that bypass p53 may be beneficial. The combination mitigated doxorubicin-induced senescence with evidence of increased apoptosis via BCL-2 family members and cleaved-caspase 3. We further demonstrate the combination significantly inhibits tumor growth in vivo. Due to bocodepsin’s favorable toxicity profile versus other HDAC inhibitors, this work supports the clinical investigation of doxorubicin and bocodepsin for metastatic TNBC.

### Electronic supplementary material

Below is the link to the electronic supplementary material.

Supplementary Material 1. (Supplemental 1A.) TNBC cell lines from Fig. 1A with corresponding p53 mutation and doxorubicin IC_50_ value. All mutations were found on https://www.cellosaurus.org/. (Supplemental 1B) Pro-apoptotic effects of DOX in combination with OKI-005. Representative plots of Annexin V/ PI in the cell lines CAL-51, MDA-MB-231, Hs 578T, and CAL-120 cell lines after 24 hours of treatment with DOX (0.5 µM), OKI-005 (0.2 µM), or combination as measured by flow cytometry. Ordinary one-way ANOVA with Tukey correction comparing single agent to combination (* = p < 0.05, ** = P < 0.01). (Supplemental 1 C) Densitometry of cleaved caspase-3 quantified by ImageJ. Cleaved caspase-3 was normalized to no drug for each treatment group along with their corresponding loading control (actin). (Supplemental 1D) β-galactosidase expression quantified by measuring β-galactosidase pixel density with ImageJ. (Supplemental 2A) Net body weight for Fig. 5 *in vivo* xenograft study. (Supplemental 2B) qRT-PCR confirming adequate KD of p53 using TaqMan gene expression assay (Applied Biosystems). (Supplemental 2 C) Pro-apoptotic effects of DOX in combination with OKI-005. Representative plots of Annexin V/ PI in the cell lines CAL-51 SCR and CAL-51 P53-10 cell lines after 24 hours of treatment with DOX (0.5 µM), OKI-005 (0.2 µM), or combination as measured by flow cytometry. Ordinary one-way ANOVA with Tukey correction comparing single agent to combination (* = p < 0.05, *** = P < 0.001). (Supplemental 2D) Percent senescent cells in CAL-51 SCR and CAL-51 P53-10 after 6-days drug treatment. Three independent fields were hand counted and the percentage was calculated based off total cells present

Supplementary Material 2

Supplementary Material 3

## Data Availability

All data generated or analyzed during this study are included in this published article [and its supplementary information files].

## Abbreviations

TNBC: Triple-negative breast cancer
ER: Estrogen receptor
PR: Progesterone receptor
HER2: Human epidermal growth factor receptor 2
DOX: Doxorubicin
TIS: Therapy-induced senescence
SASP: Senescence-associated secretory phenotype
HDAC(i): Histone deacetylase (inhibitor)
KD: Knockdown
SCR: Scramble
β-gal: β-galactosidase
