# Supplementary material for: Overcoming doxorubicin resistance in triple-negative breast cancer using the class I-targeting HDAC inhibitor bocodepsin/OKI-179 to promote apoptosis
Source: Breast Cancer Res. 2024 Mar 1;26:35. doi: 10.1186/s13058-024-01799-5 (PMC10908182; doi:10.1186/s13058-024-01799-5)
Supplement: Supplementary file 1 — Supplementary Material 1. (Supplemental 1A.) TNBC cell lines from Fig. 1A with corresponding p53 mutation and doxorubicin IC50 value. All mutations were found on https://www.cellosaurus.org/. (Supplemental 1B) Pro-apoptotic effects of DOX in combination with OKI-005. Representative plots of Annexin V/ PI in the cell lines CAL-51, MDA-MB-231, Hs 578T, and CAL-120 cell lines after 24 hours of treatment with DOX (0.5 µM), OKI-005 (0.2 µM), or combination as measured by flow cytometry. Ordinary one-way ANOVA with Tukey correction comparing single agent to combination (* = p < 0.05, ** = P < 0.01). (Supplemental 1 C) Densitometry of cleaved caspase-3 quantified by ImageJ. Cleaved caspase-3 was normalized to no drug for each treatment group along with their corresponding loading control (actin). (Supplemental 1D) β-galactosidase expression quantified by measuring β-galactosidase pixel density with ImageJ. (Supplemental 2A) Net body weight for Fig. 5 in vivo xenograft study. (Supplemental 2B) qRT-PCR confirming adequate KD of p53 using TaqMan gene expression assay (Applied Biosystems). (Supplemental 2 C) Pro-apoptotic effects of DOX in combination with OKI-005. Representative plots of Annexin V/ PI in the cell lines CAL-51 SCR and CAL-51 P53-10 cell lines after 24 hours of treatment with DOX (0.5 µM), OKI-005 (0.2 µM), or combination as measured by flow cytometry. Ordinary one-way ANOVA with Tukey correction comparing single agent to combination (* = p < 0.05, *** = P < 0.001). (Supplemental 2D) Percent senescent cells in CAL-51 SCR and CAL-51 P53-10 after 6-days drug treatment. Three independent fields were hand counted and the percentage was calculated based off total cells present [file 13058_2024_1799_MOESM1_ESM.pdf]

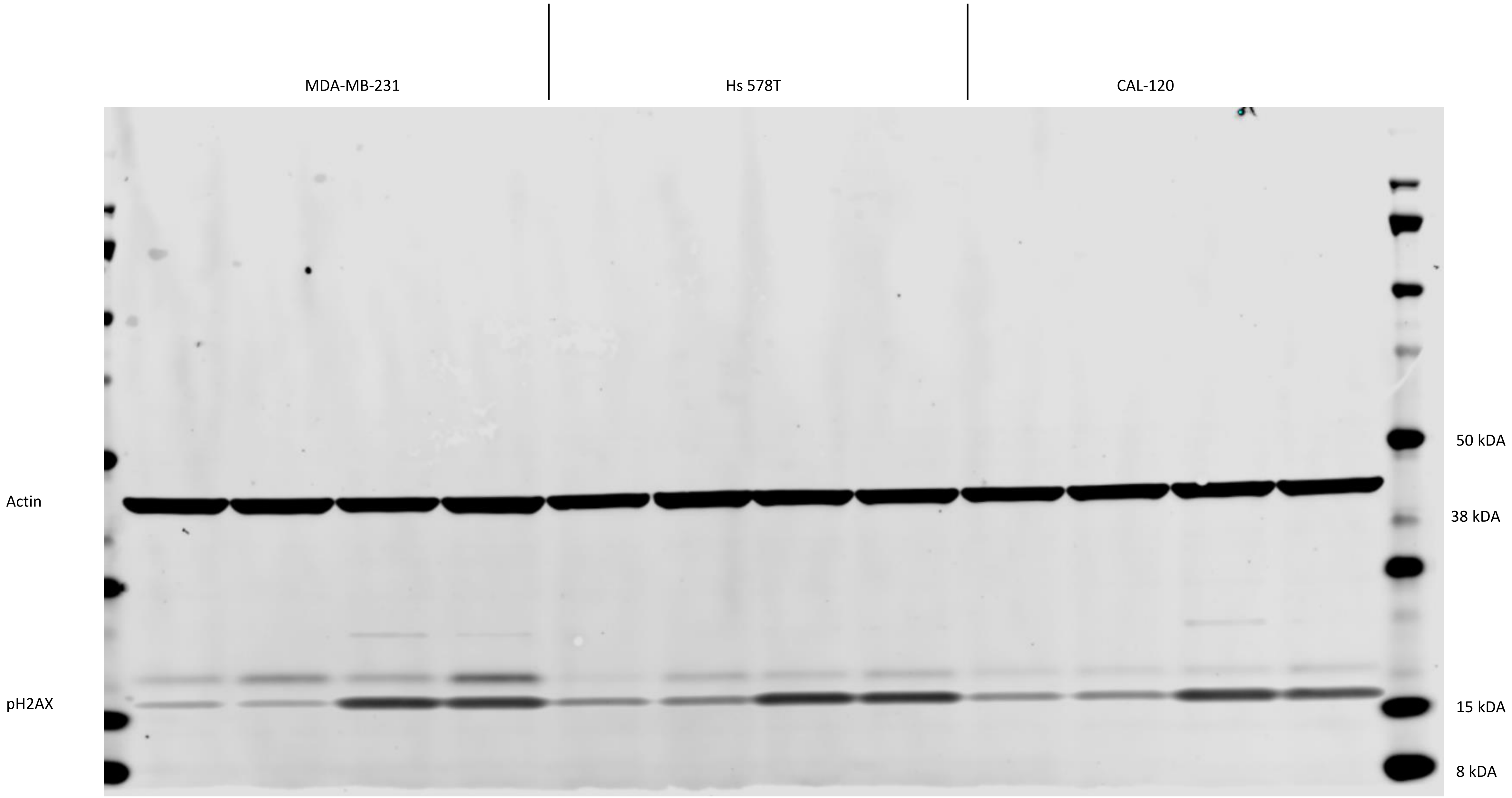
MDA-MB-231
Hs 578T
CAL-120
Actin
pH2AX
50 kDA
38 kDA
15 kDA
8 kDA

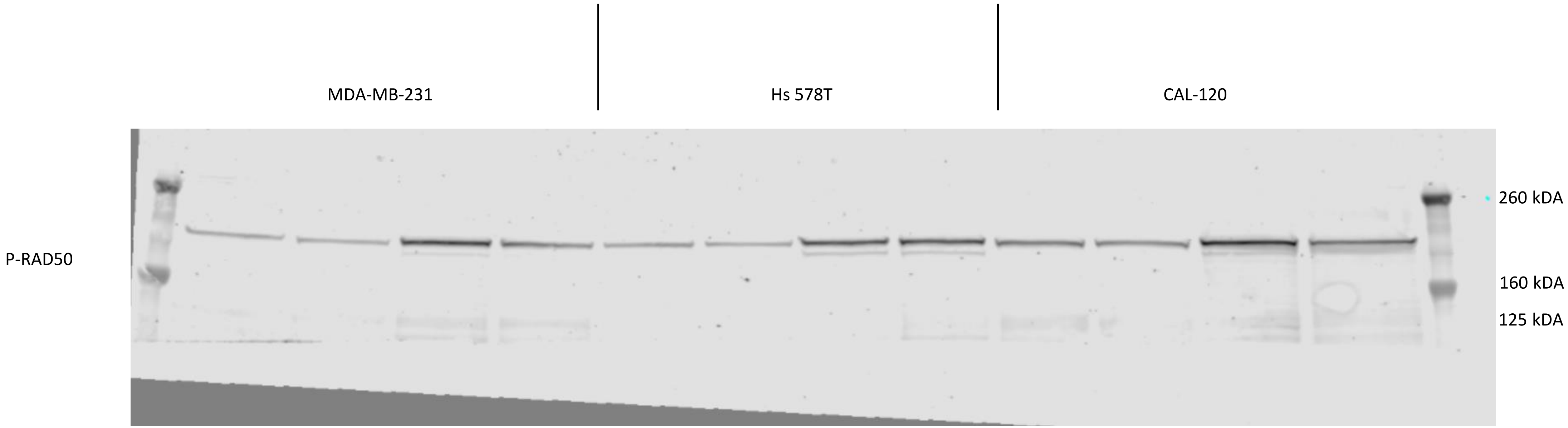
MDA-MB-231
Hs 578T
CAL-120
P-RAD50
260 kDA
160 kDA
125 kDA

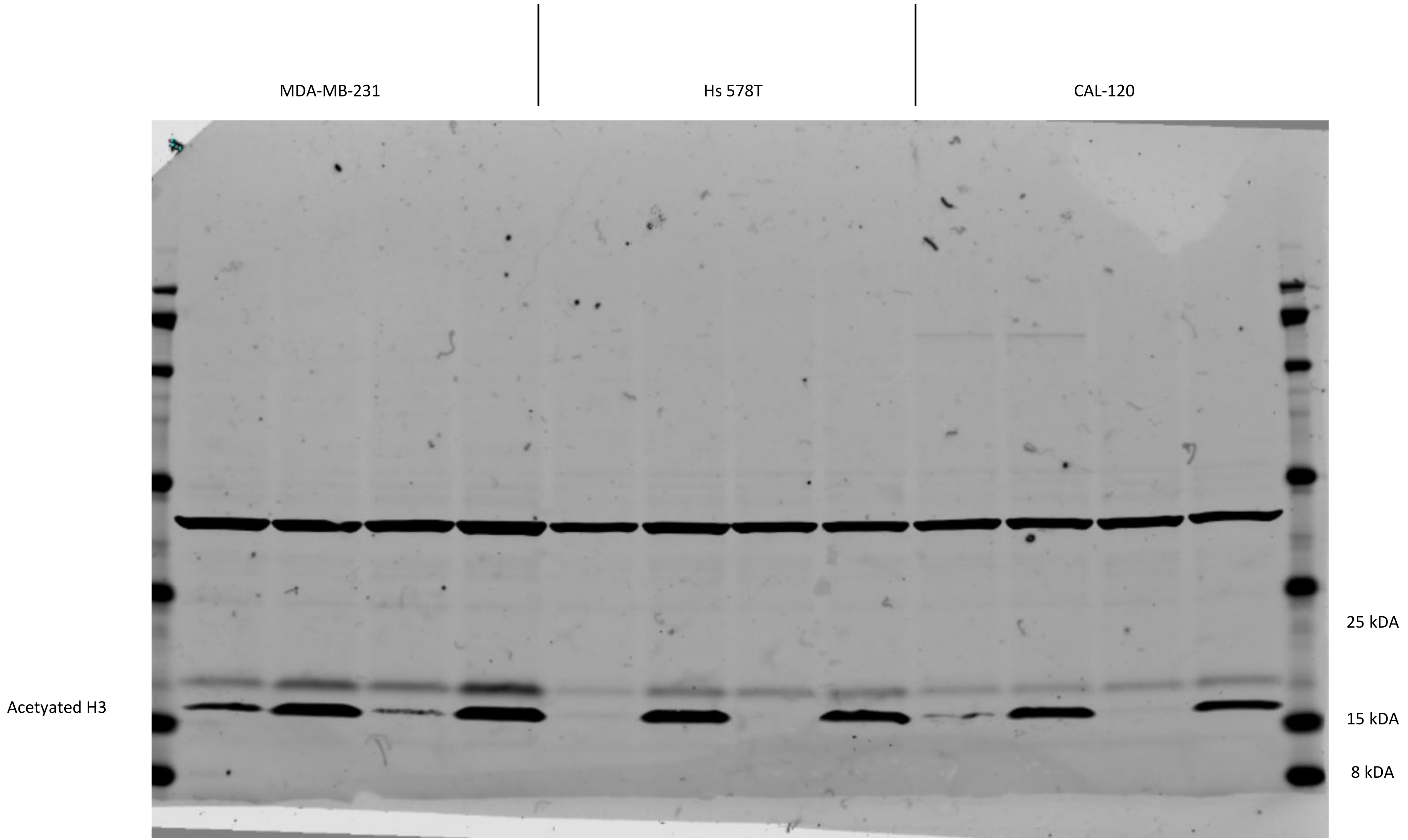
MDA-MB-231
Hs 578T
CAL-120
25 kDA
15 kDA
8 kDA
Acetyated H3

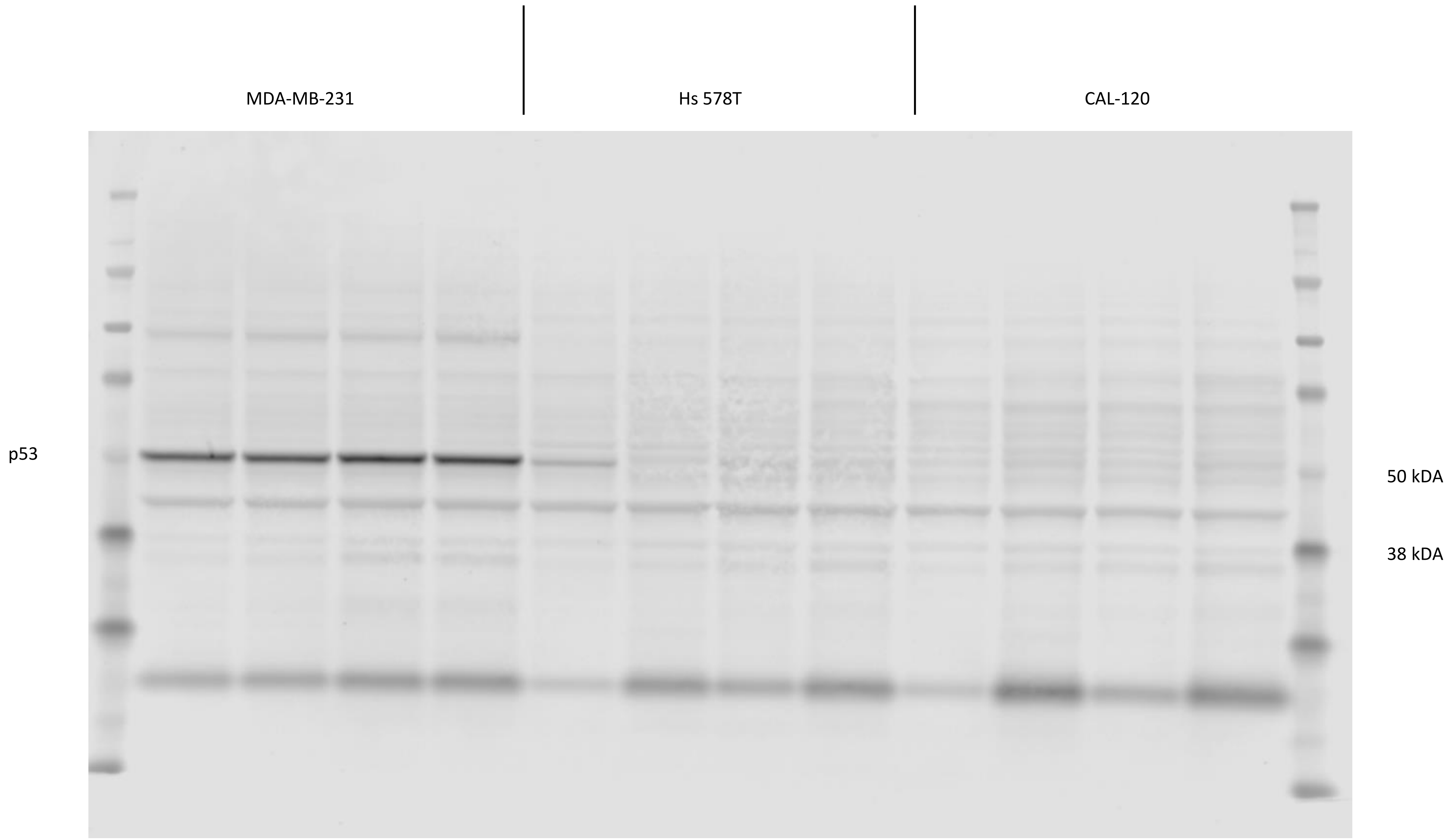
MDA-MB-231
Hs 578T
CAL-120
p53
50 kDA
38 kDA

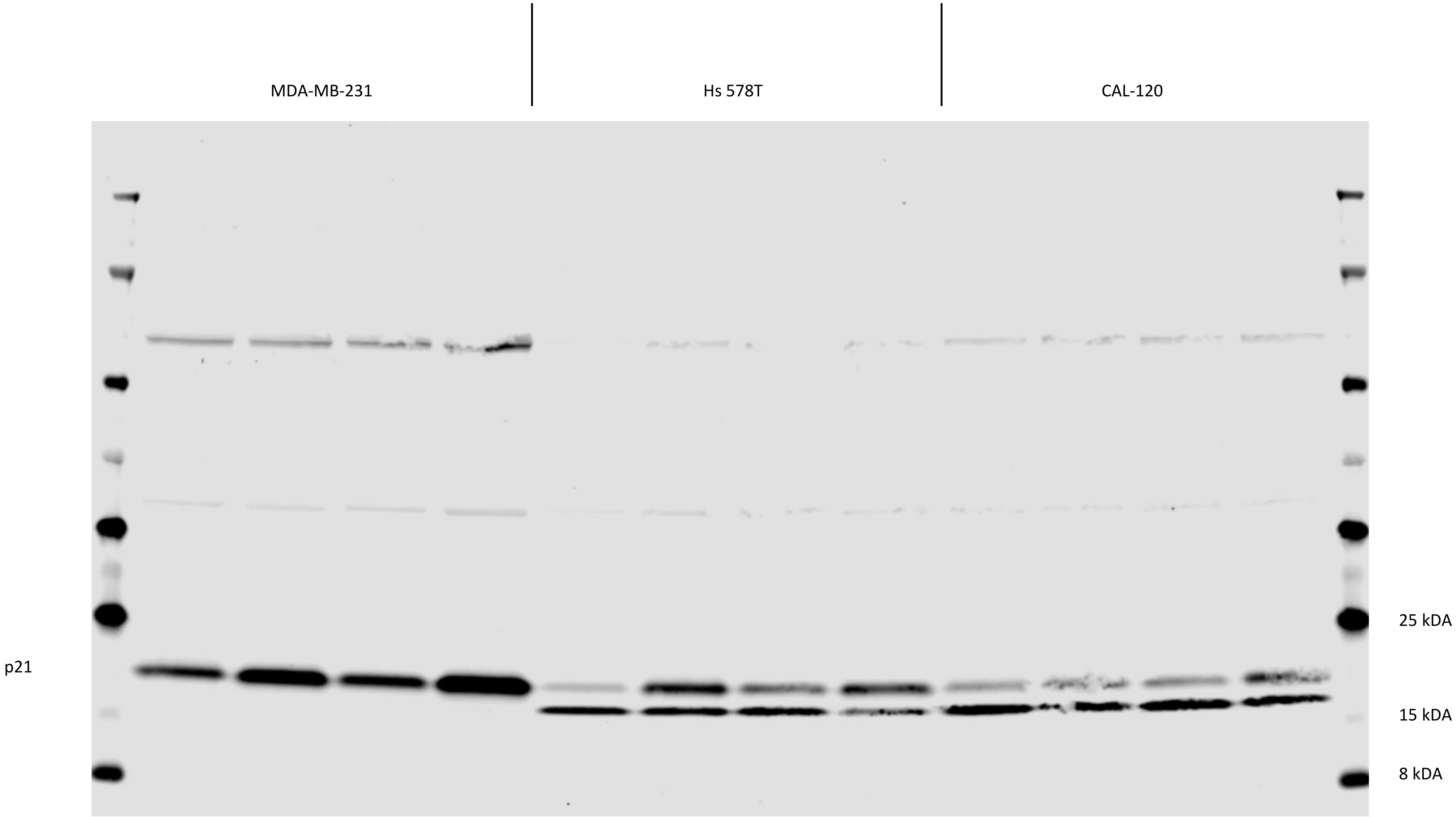
MDA-MB-231
Hs 578T
CAL-120
p21
25 kDA
15 kDA
8 kDA

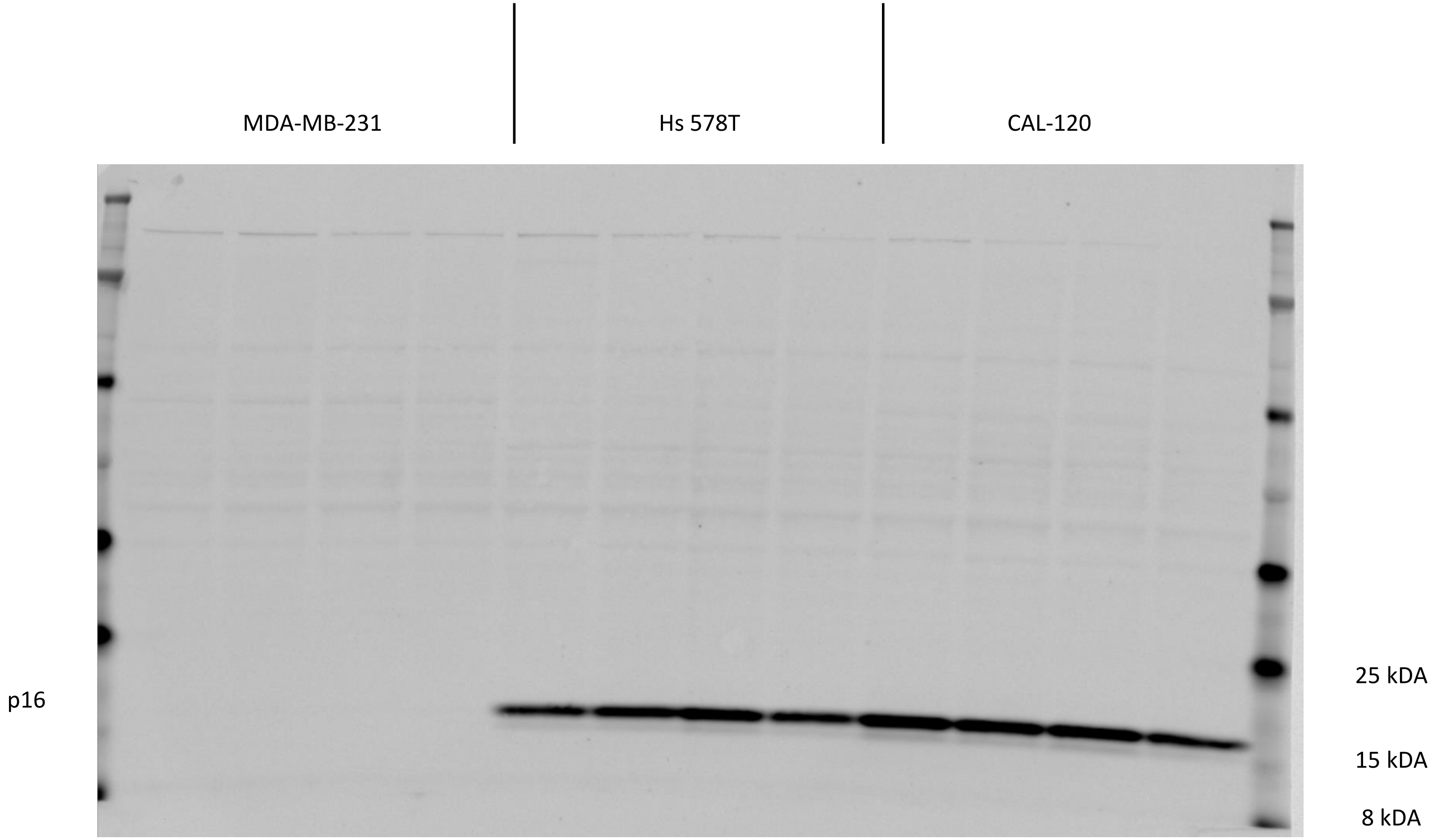

MDA-MB-231
Hs 578T
CAL-120
p16
25 kDA
15 kDA
8 kDA

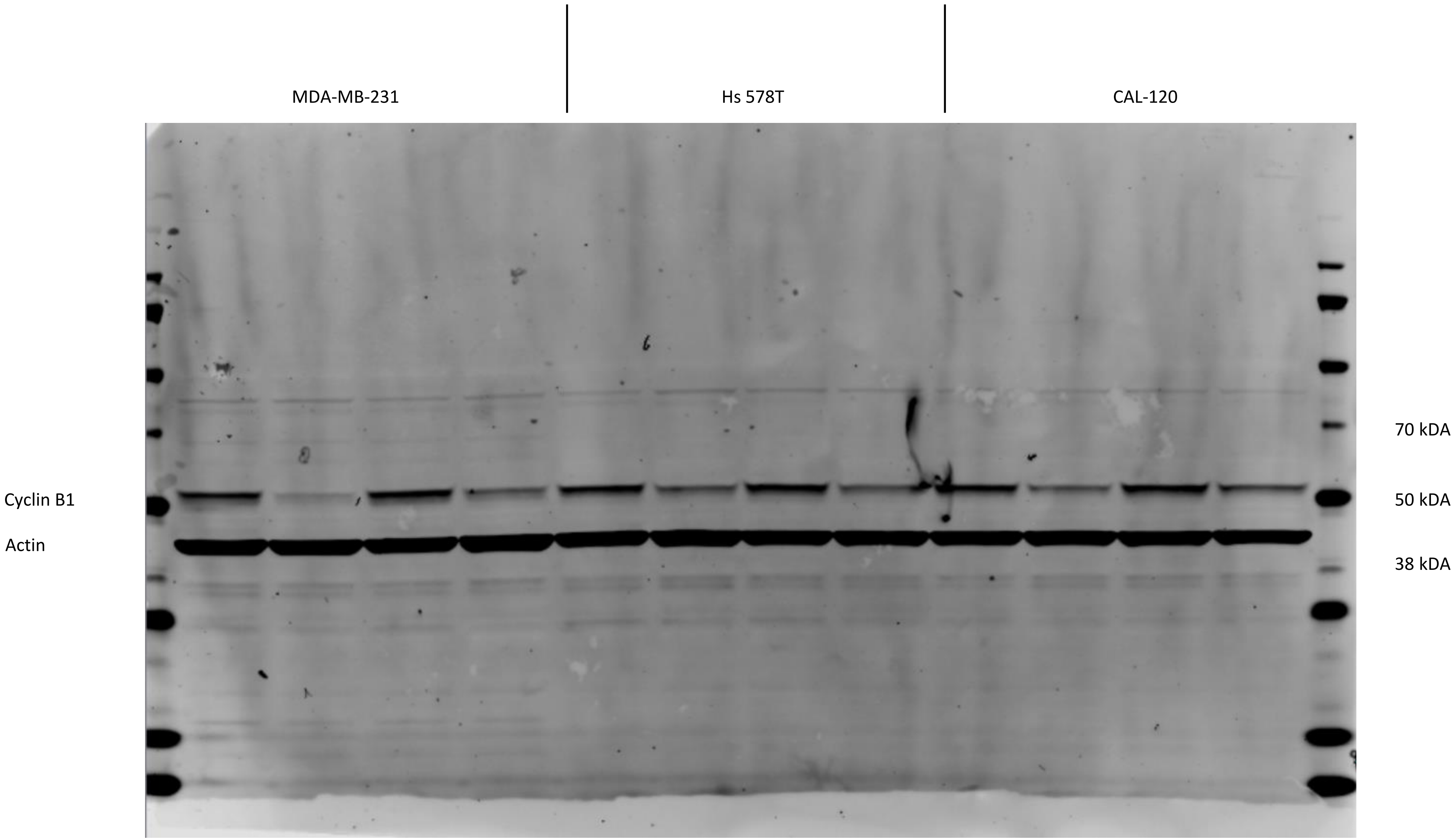
MDA-MB-231
Hs 578T
CAL-120
Cyclin B1
Actin
70 kDA
50 kDA
38 kDA

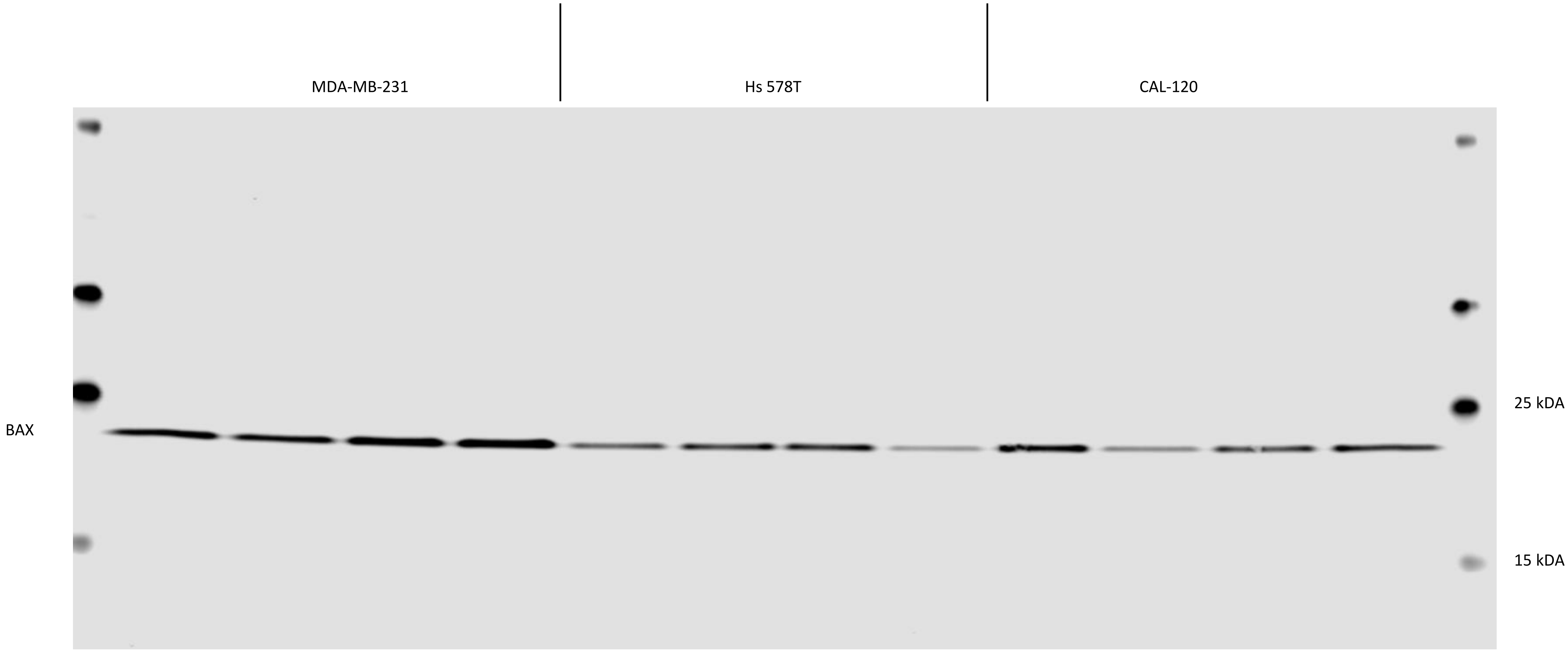

MDA-MB-231
Hs 578T
CAL-120
BAX
25 kDA
15 kDA

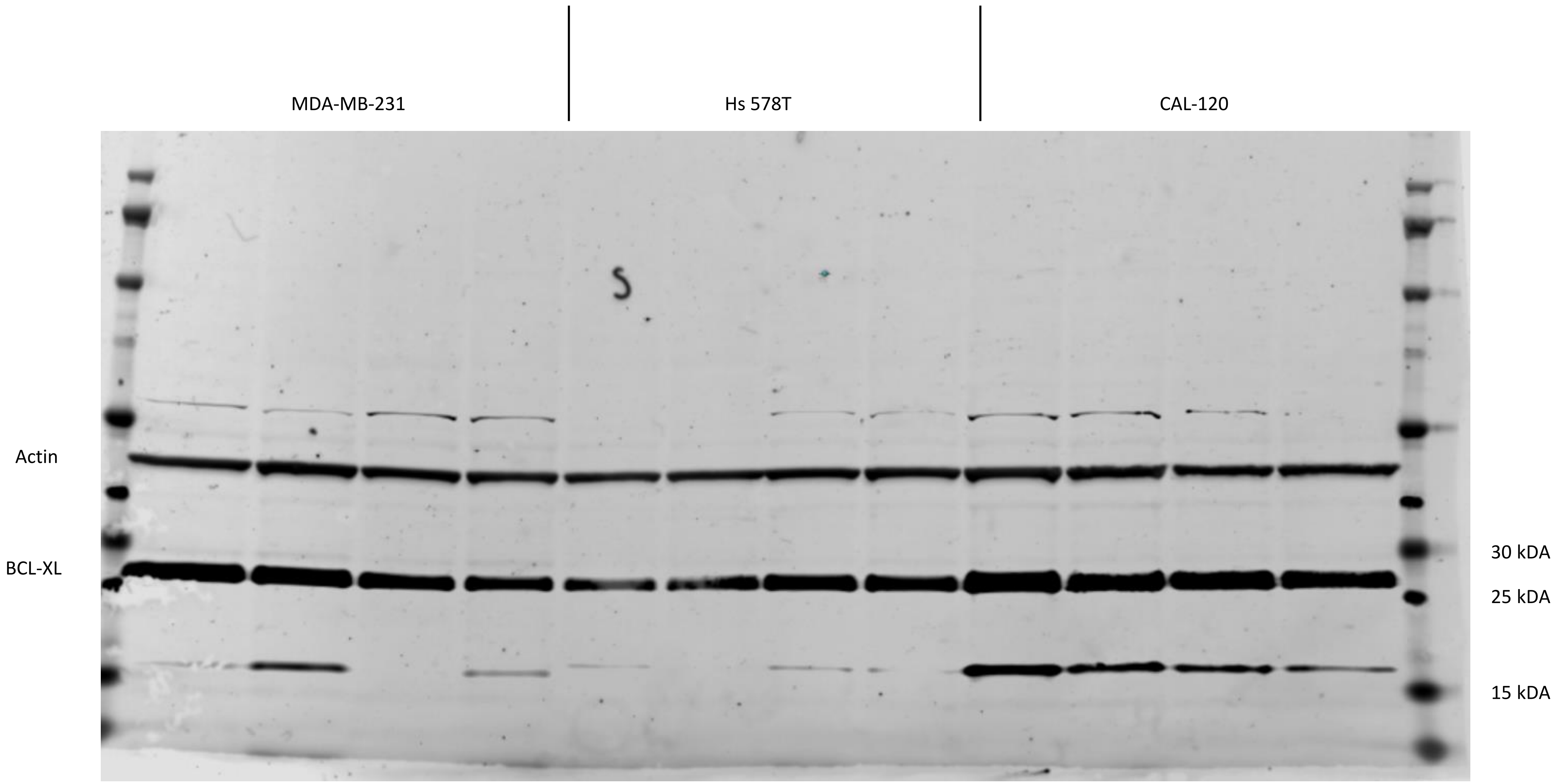
MDA-MB-231
Hs 578T
CAL-120
Actin
BCL-XL
30 kDA
25 kDA
15 kDA

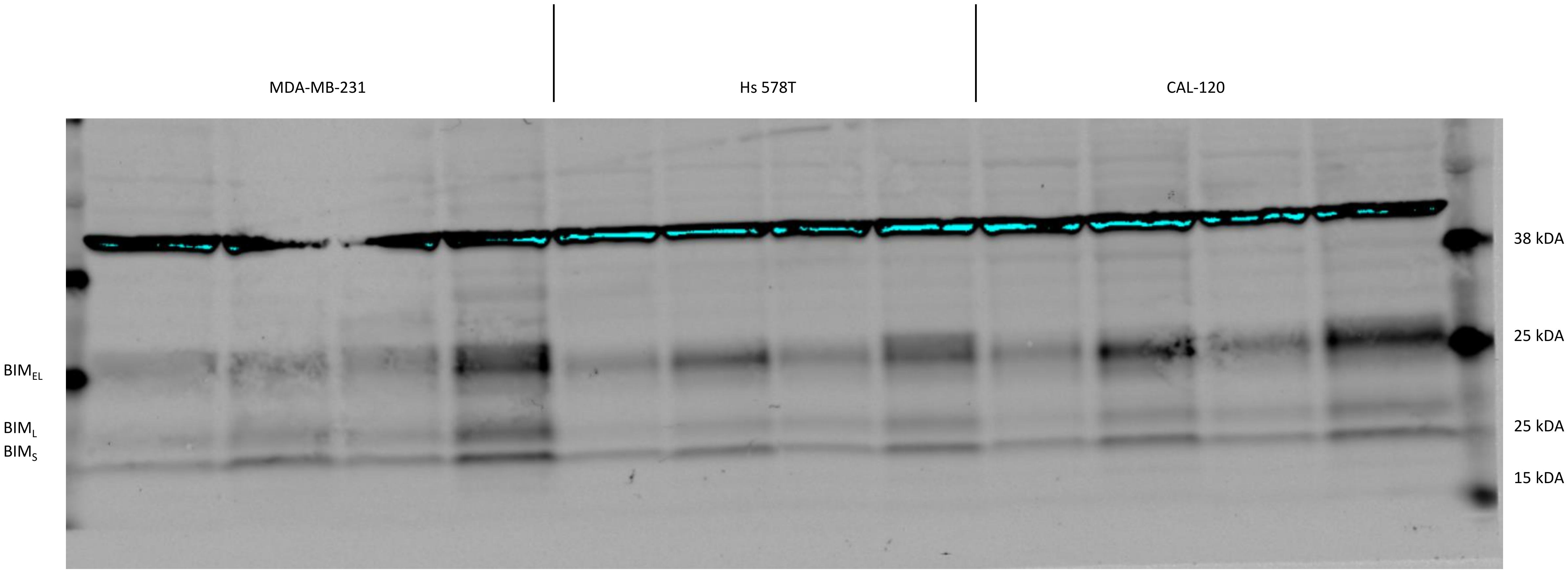

MDA-MB-231
Hs 578T
CAL-120
38 kDA
25 kDA
25 kDA
15 kDA
BIM$_{EL}$
BIM$_{L}$
BIM$_{S}$

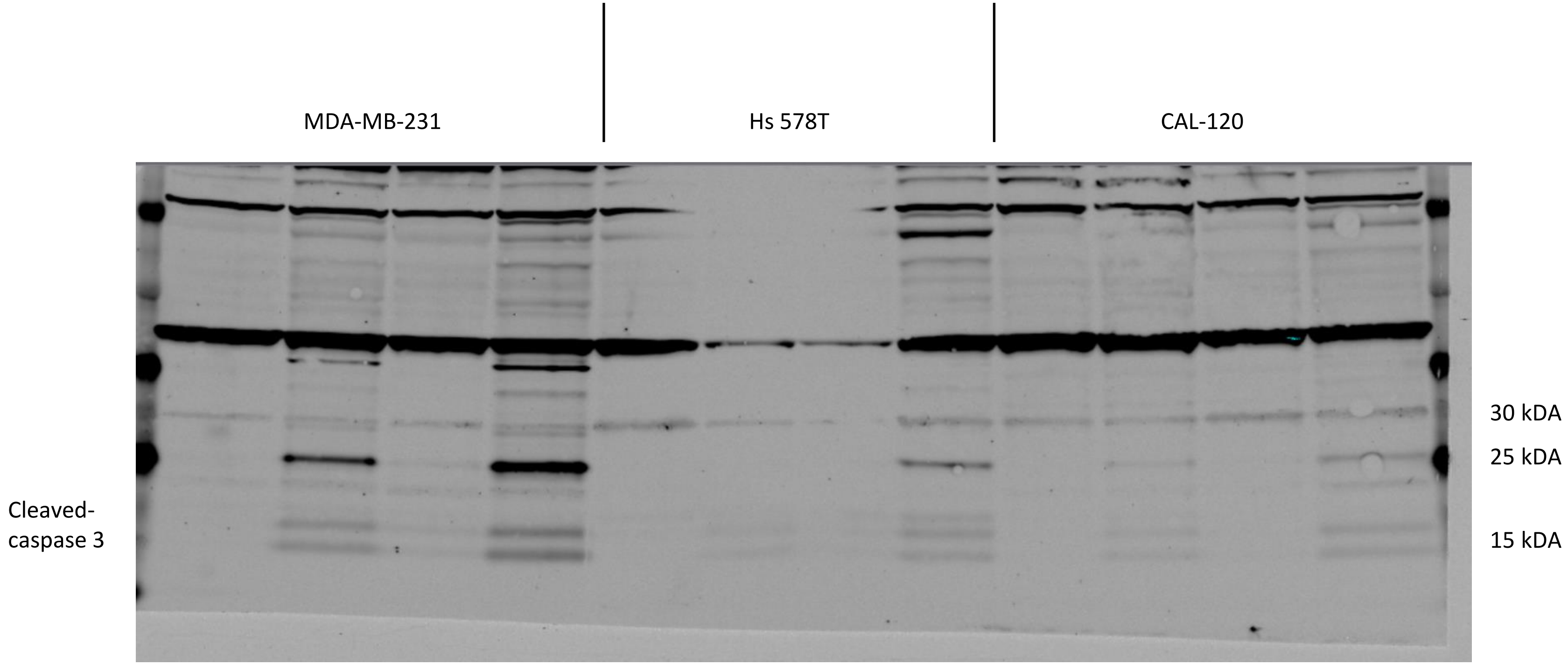
MDA-MB-231
Hs 578T
CAL-120
30 kDA
25 kDA
15 kDA
Cleaved-
caspase 3

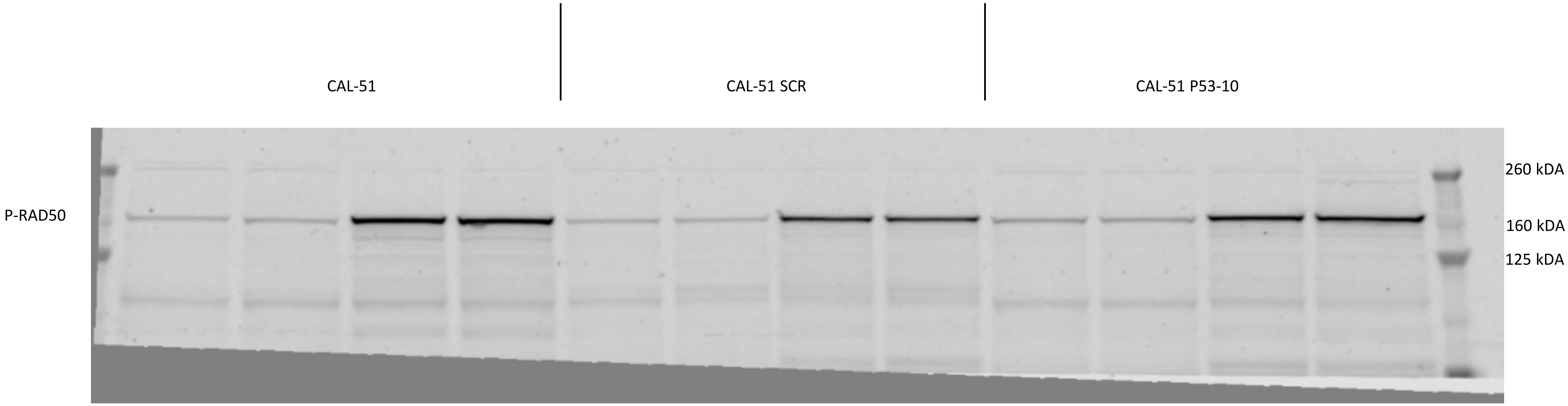

CAL-51
CAL-51 SCR
CAL-51 P53-10
P-RAD50
260 kDA
160 kDA
125 kDA

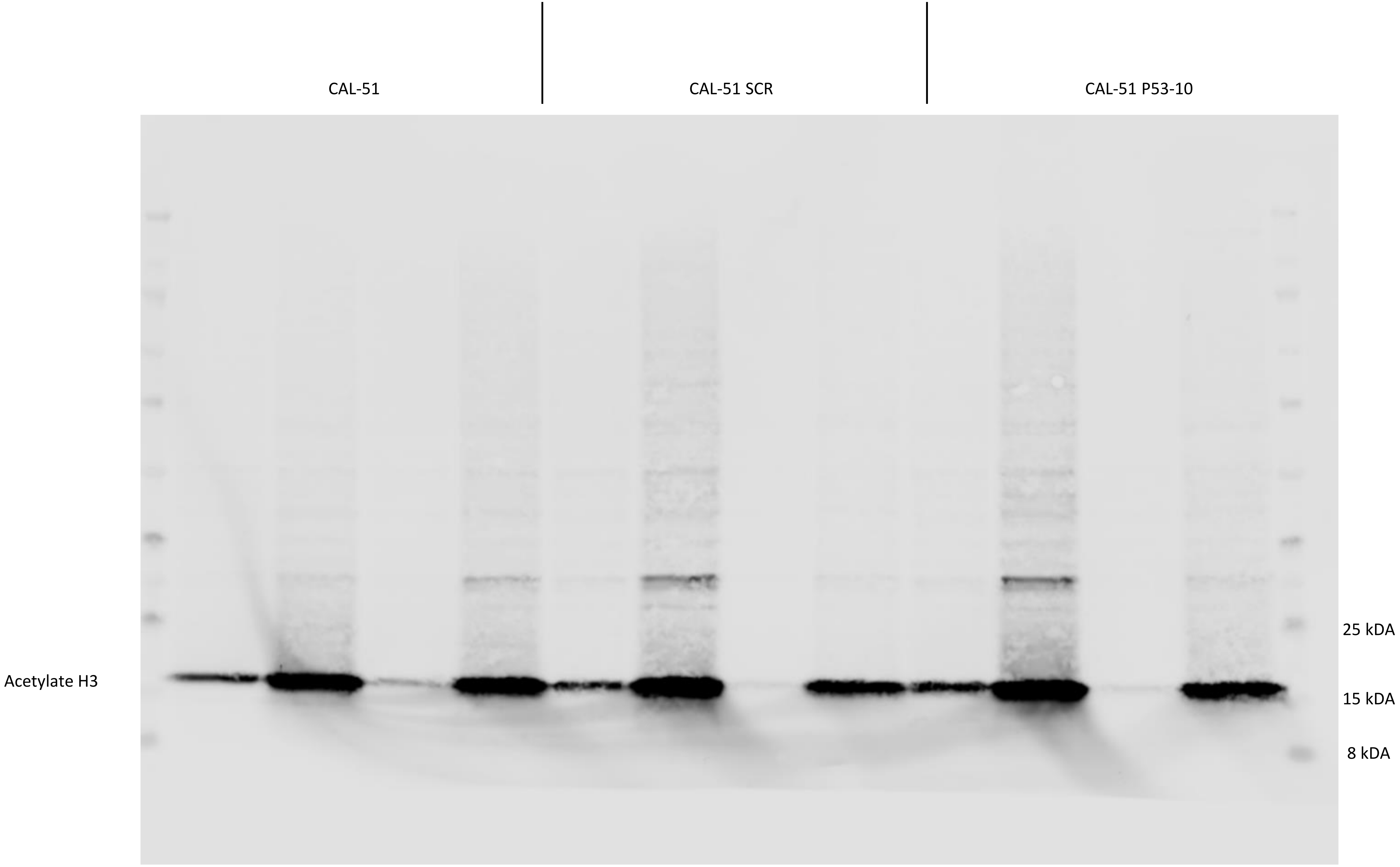

CAL-51
CAL-51 SCR
CAL-51 P53-10
Acetylate H3
25 kDA
15 kDA
8 kDA

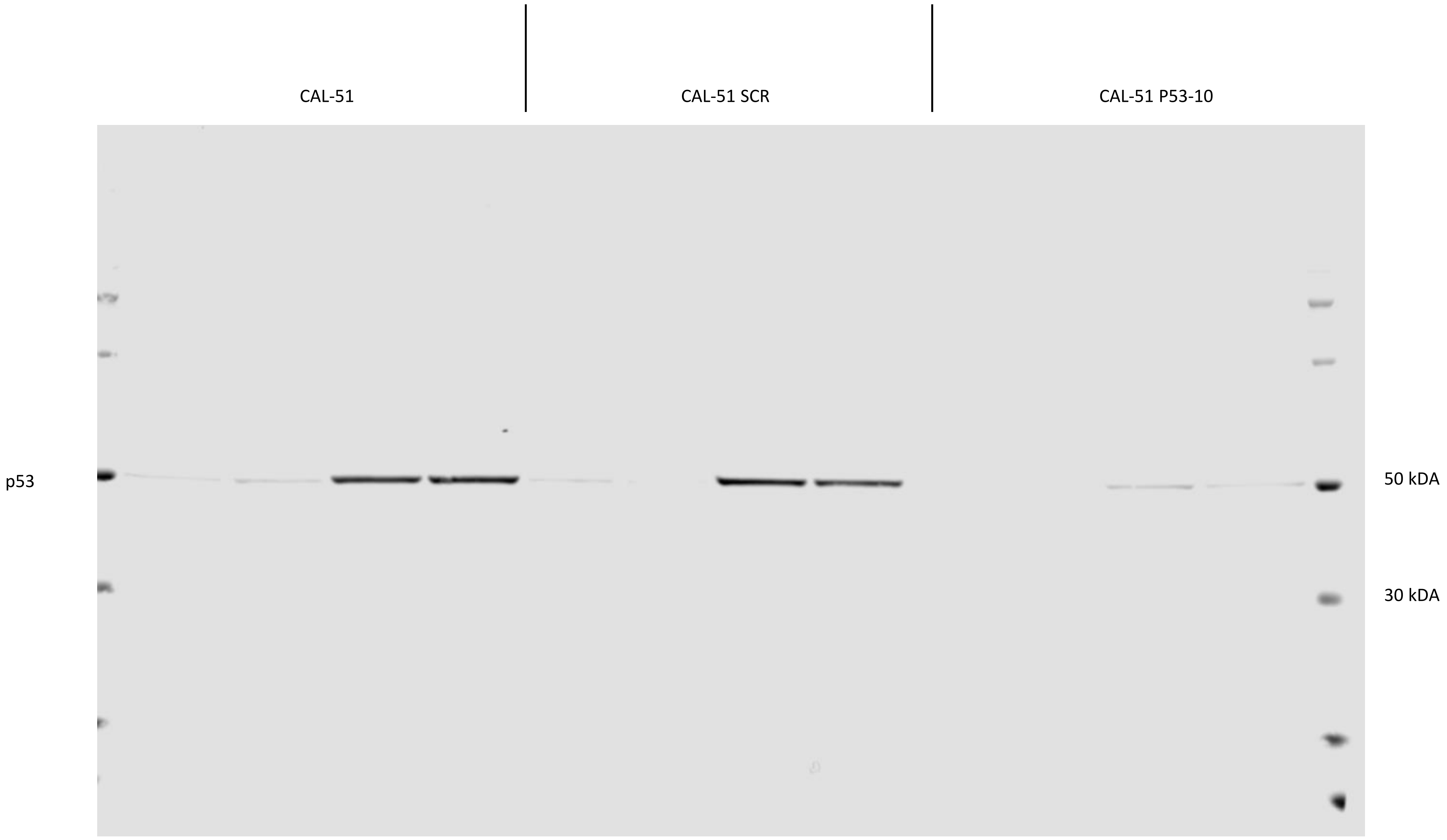

CAL-51
CAL-51 SCR
CAL-51 P53-10
p53
50 kDA
30 kDA

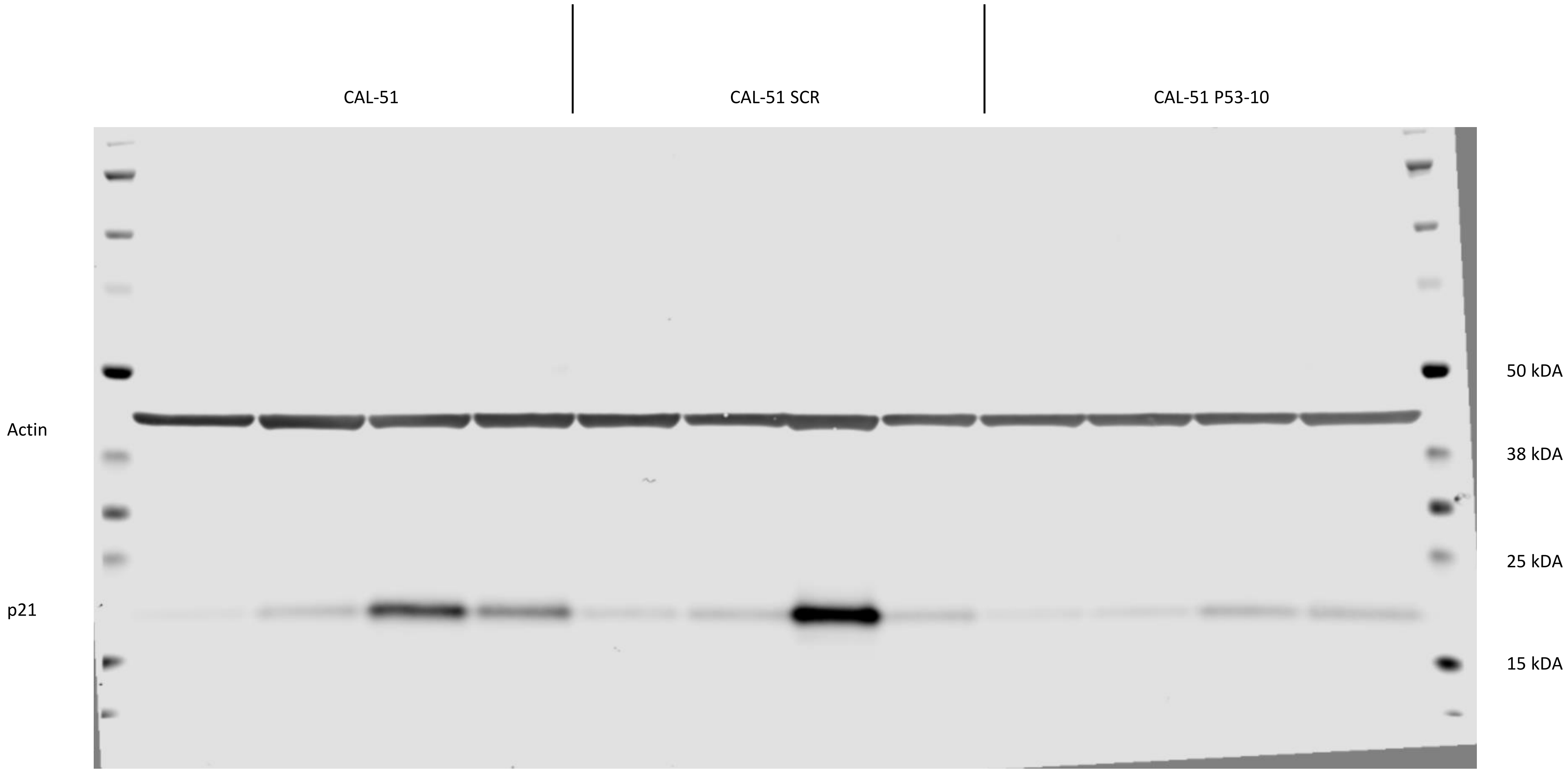
CAL-51
CAL-51 SCR
CAL-51 P53-10
Actin
p21
50 kDA
38 kDA
25 kDA
15 kDA

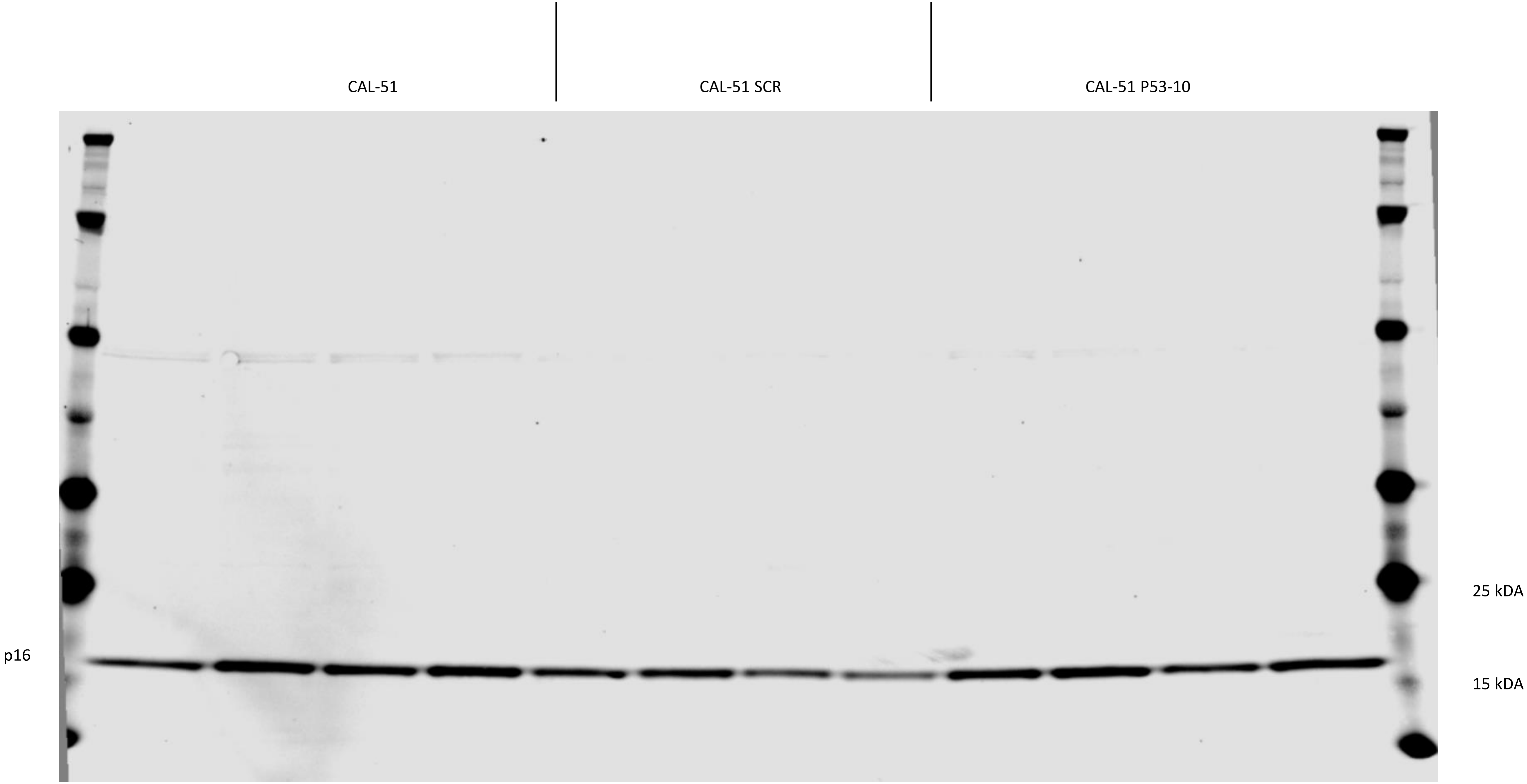
CAL-51
CAL-51 SCR
CAL-51 P53-10
p16
25 kDA
15 kDA

CAL-51 | CAL-51 SCR | CAL-51 P53-10

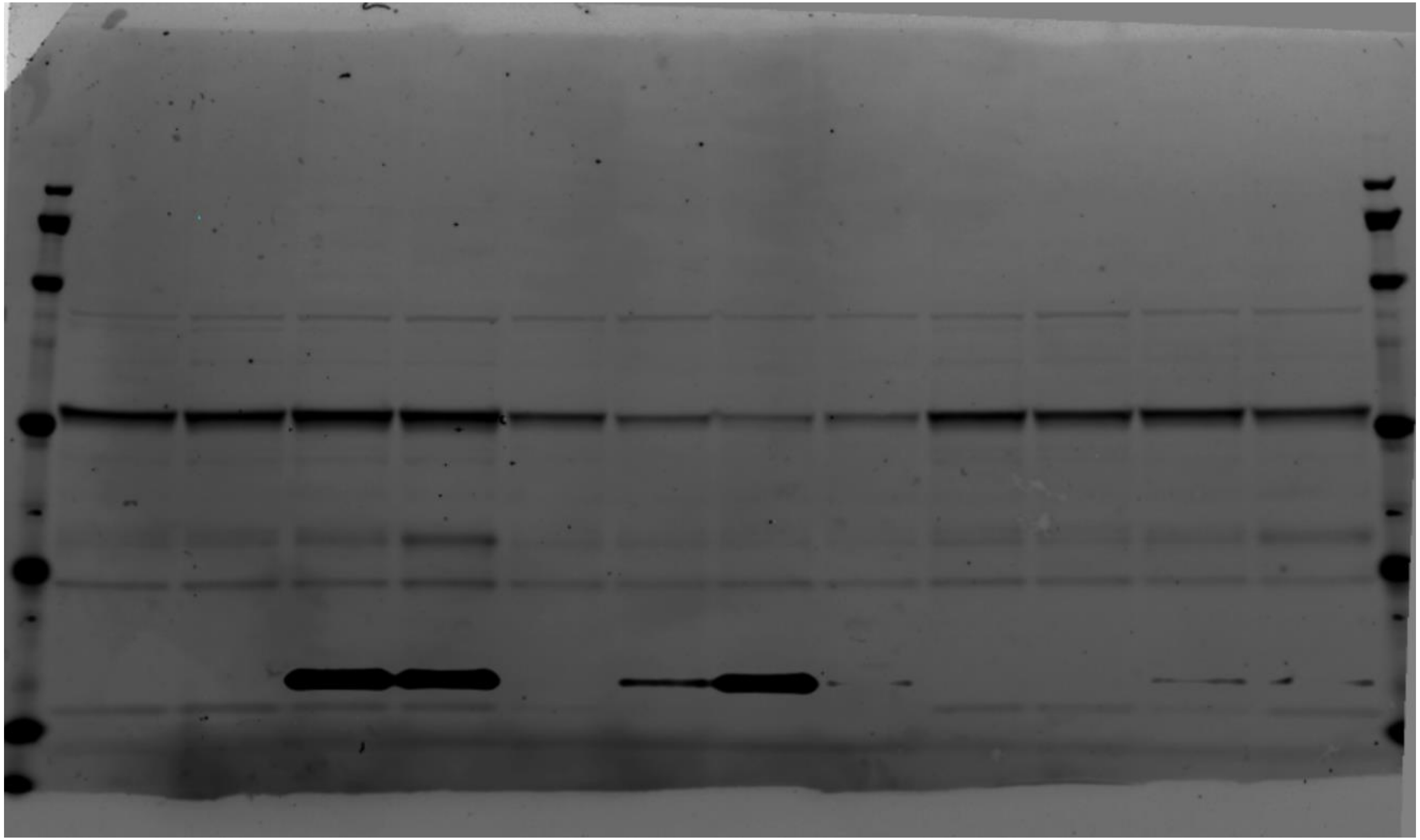

Cyclin B1

70 kDA

50 kDA

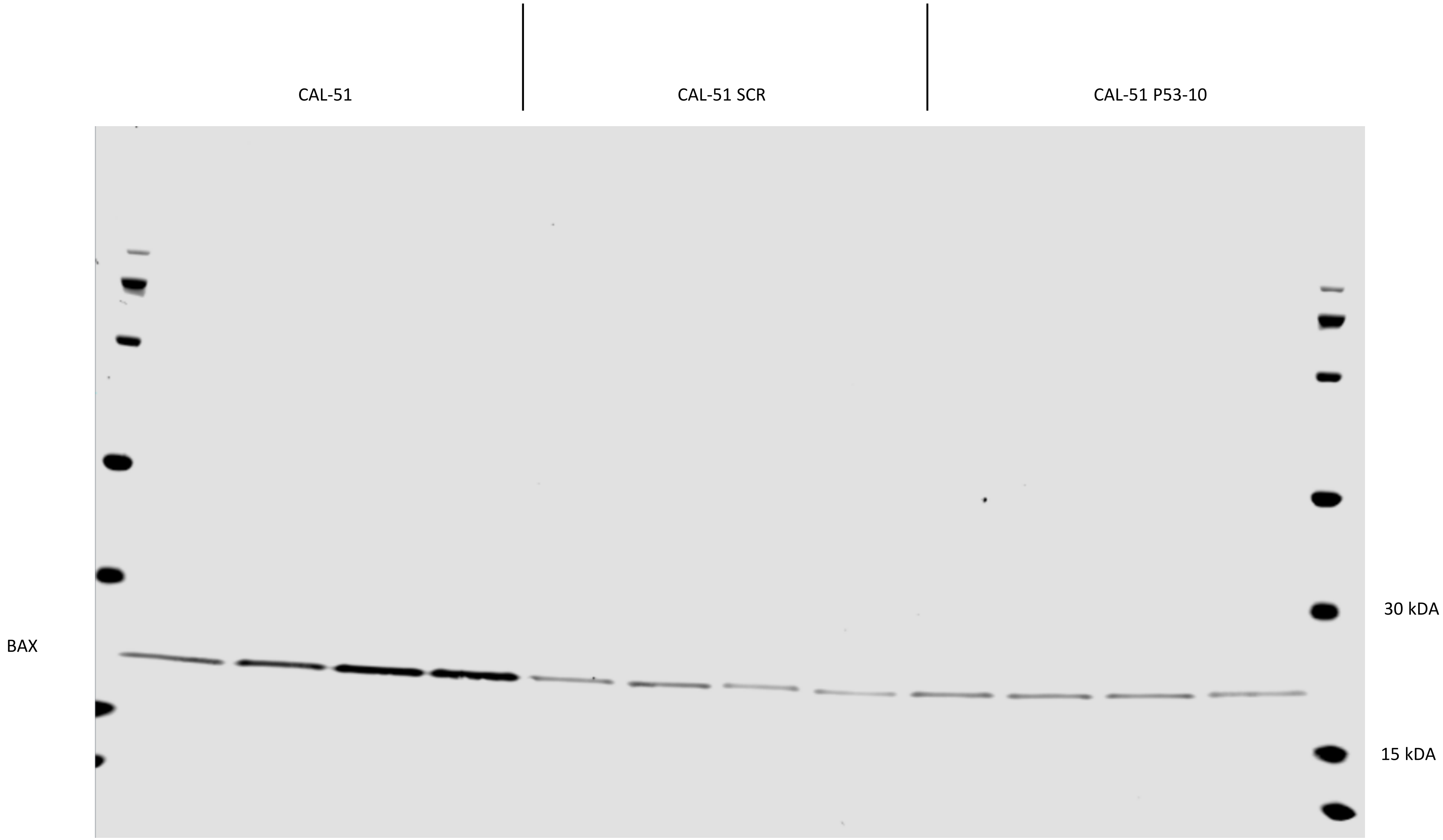
CAL-51
CAL-51 SCR
CAL-51 P53-10
BAX
30 kDA
15 kDA

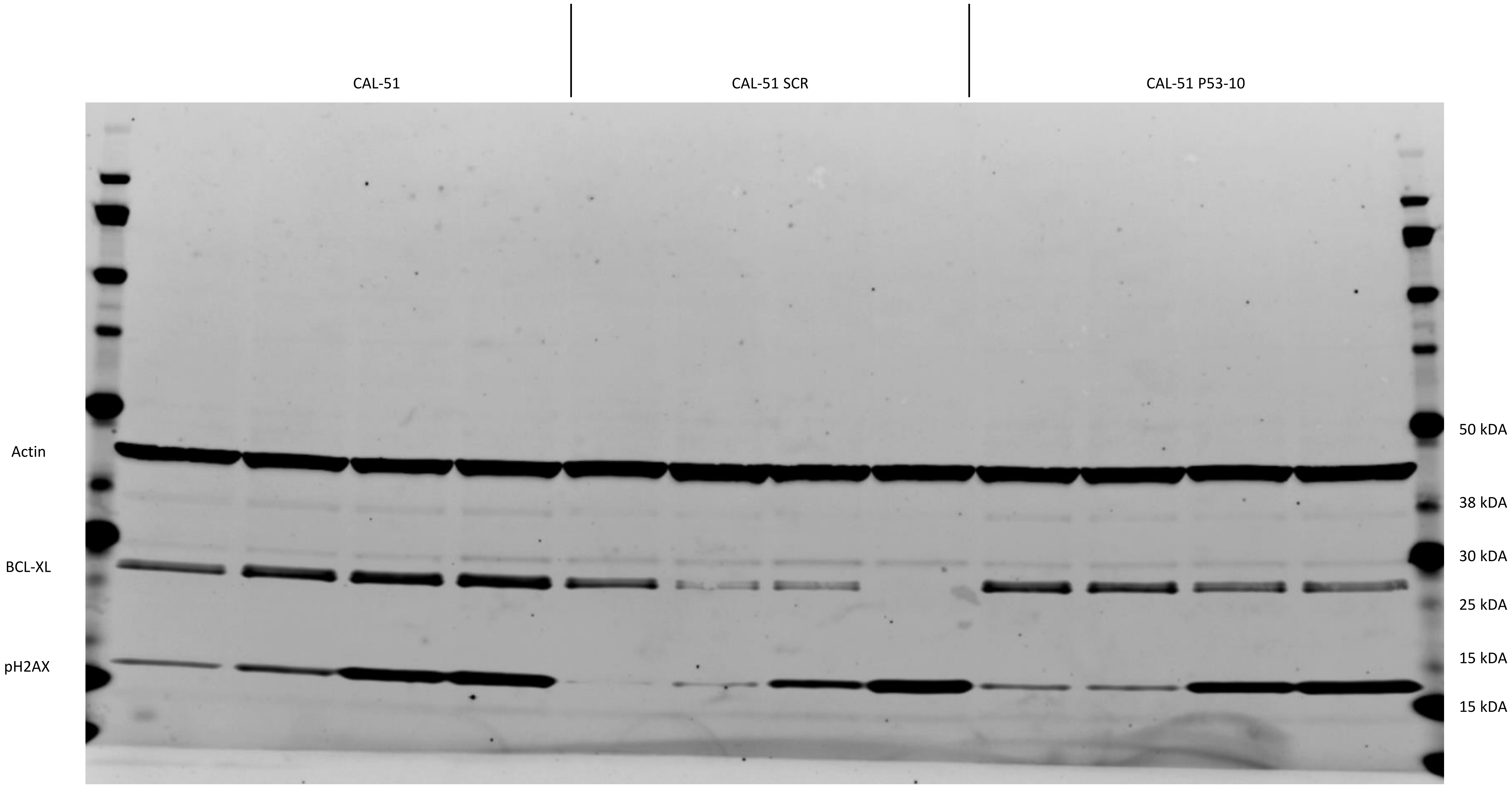

CAL-51
CAL-51 SCR
CAL-51 P53-10
Actin
BCL-XL
pH2AX
50 kDA
38 kDA
30 kDA
25 kDA
15 kDA
15 kDA

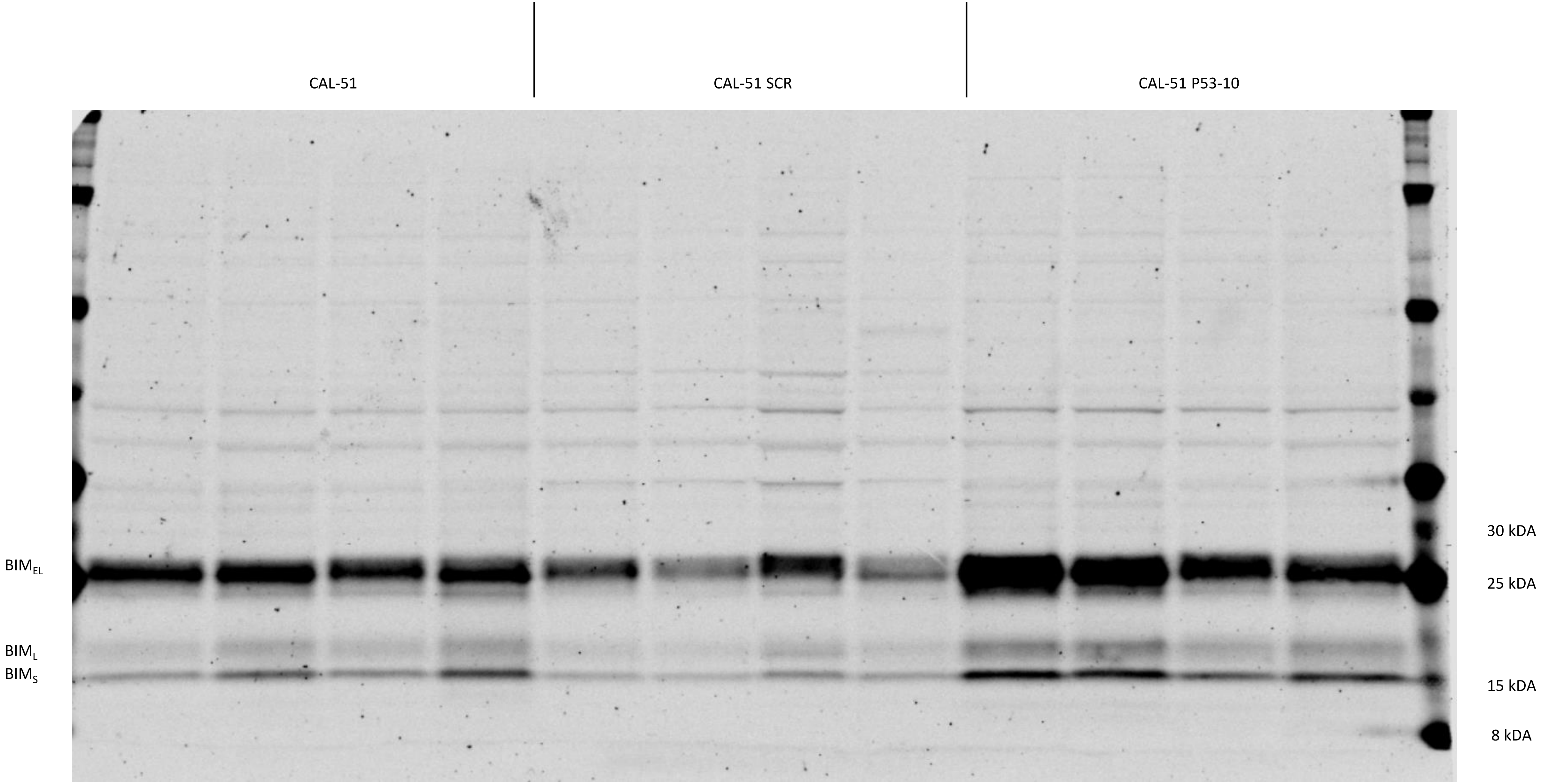
CAL-51
CAL-51 SCR
CAL-51 P53-10
BIM_{EL}
BIM_L
BIM_S
30 kDA
25 kDA
15 kDA
8 kDA

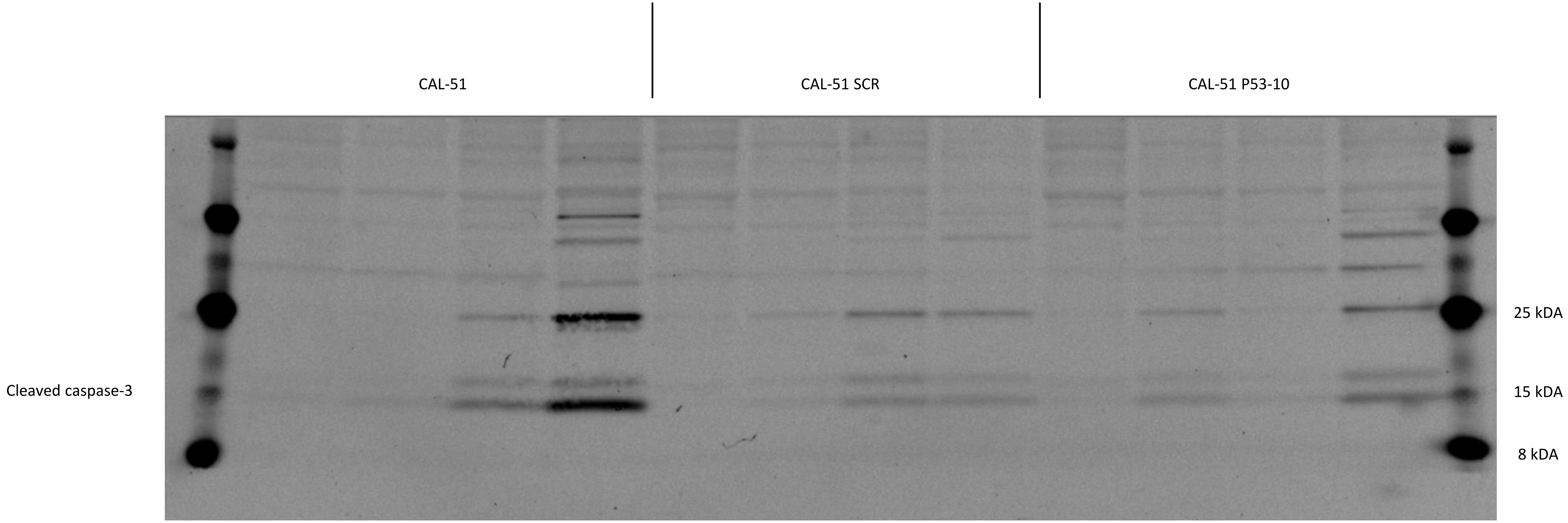
CAL-51
CAL-51 SCR
CAL-51 P53-10
Cleaved caspase-3
25 kDA
15 kDA
8 kDA
