# Supplementary figures and images for: Overcoming doxorubicin resistance in triple-negative breast cancer using the class I-targeting HDAC inhibitor bocodepsin/OKI-179 to promote apoptosis
Source: Breast Cancer Res. 2024 Mar 1;26:35. doi: 10.1186/s13058-024-01799-5 (PMC10908182; doi:10.1186/s13058-024-01799-5)

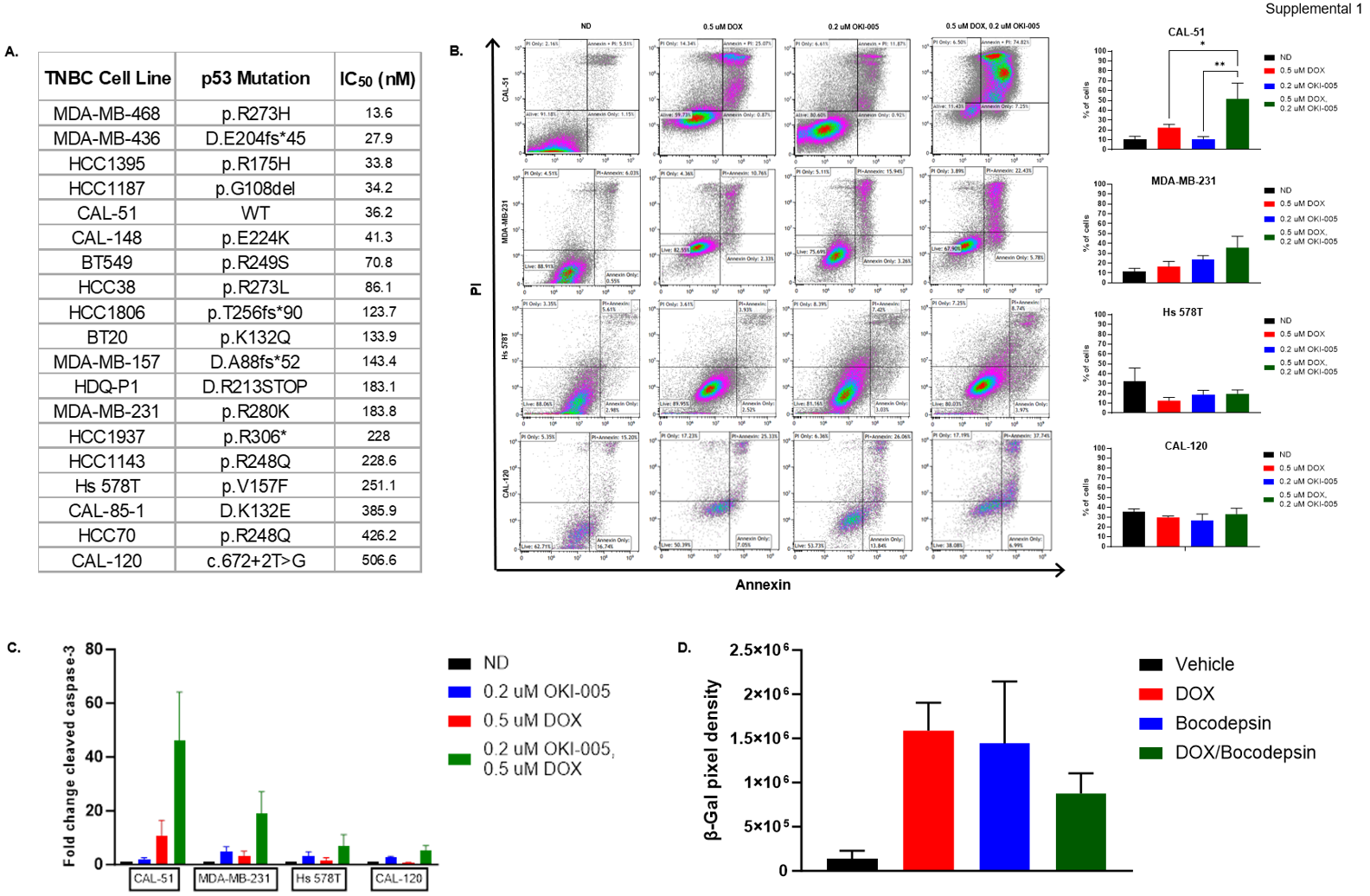

Supplement: Supplementary file 2 — Supplementary Material 2 [file 13058_2024_1799_MOESM2_ESM.tif]

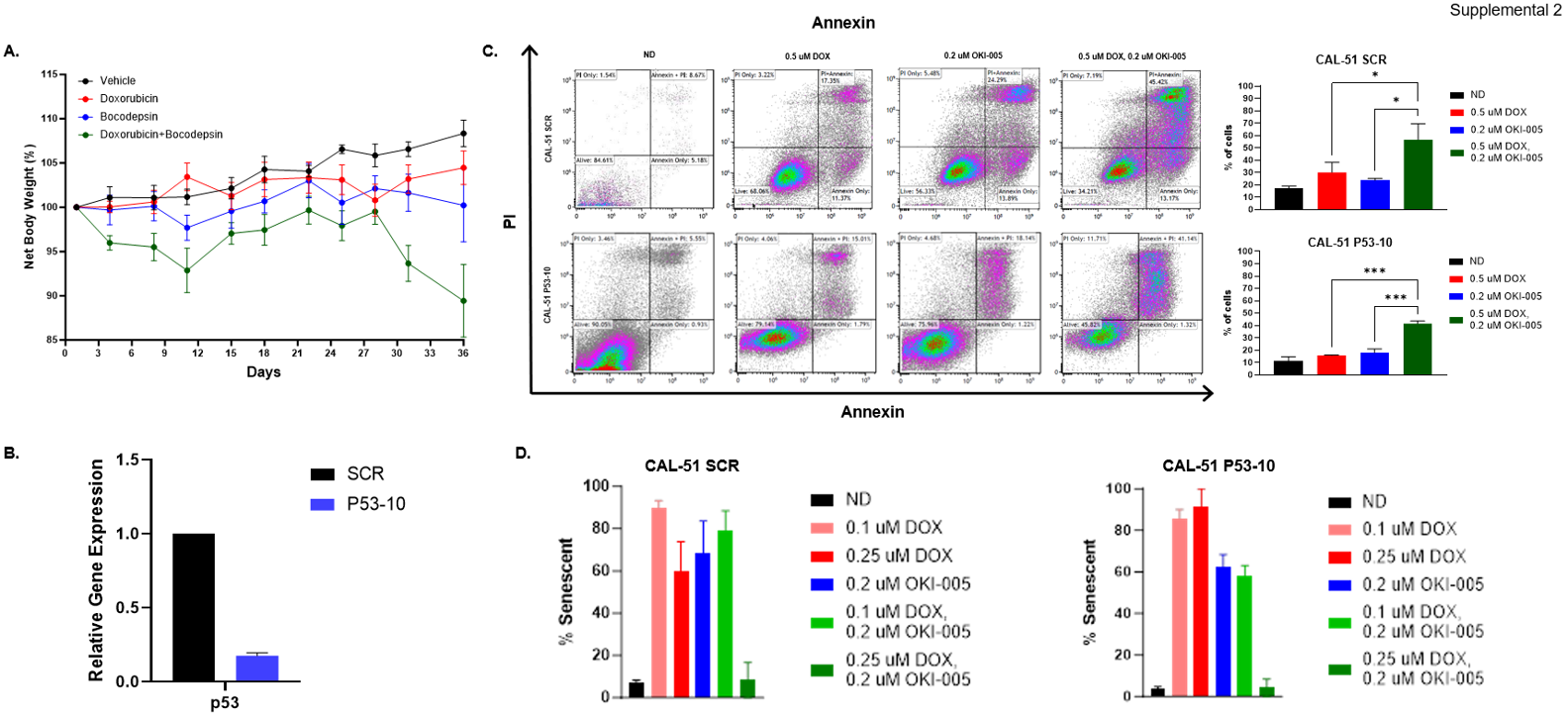

Supplement: Supplementary file 3 — Supplementary Material 3 [file 13058_2024_1799_MOESM3_ESM.png]
